# Expression capable library for studies of *Neisseria gonorrhoeae*, version 1.0

**DOI:** 10.1186/1471-2180-5-50

**Published:** 2005-09-01

**Authors:** Thomas Brettin, Michael R Altherr, Ying Du, Roxie M Mason, Alexandra Friedrich, Laura Potter, Chris Langford, Thomas J Keller, Jason Jens, Heather Howie, Nathan J Weyand, Susan Clary, Kimberly Prichard, Susi Wachocki, Erica Sodergren, Joseph P Dillard, George Weinstock, Magdalene So, Cindy Grove Arvidson

**Affiliations:** 1 Bioscience Division, Los Alamos National Laboratory, Los Alamos, NM 87545, USA; 2 Department of Microbiology and Molecular Genetics, Michigan State University, East Lansing, MI 48824-4320, USA; 3 Department of Molecular Microbiology and Immunology, Oregon Health and Science University, Portland, OR 97201-3098, USA; 4 Human Genome Sequencing Center, Baylor College of Medicine, Houston TX 77030, USA; 5 Department of Medical Microbiology and Immunology, University of Wisconsin Medical School, Madison, WI 53706, USA; 6 Leicester Warwick Medical School, University of Warwick, Coventry, UK

## Abstract

**Background:**

The sexually transmitted disease, gonorrhea, is a serious health problem in developed as well as in developing countries, for which treatment continues to be a challenge. The recent completion of the genome sequence of the causative agent, *Neisseria gonorrhoeae*, opens up an entirely new set of approaches for studying this organism and the diseases it causes. Here, we describe the initial phases of the construction of an expression-capable clone set representing the protein-coding ORFs of the gonococcal genome using a recombination-based cloning system.

**Results:**

The clone set thus far includes 1672 of the 2250 predicted ORFs of the *N. gonorrhoeae *genome, of which 1393 (83%) are sequence-validated. Included in this set are 48 of the 61 ORFs of the gonococcal genetic island of strain MS11, not present in the sequenced genome of strain FA1090. L-arabinose-inducible glutathione-S-transferase (GST)-fusions were constructed from random clones and each was shown to express a fusion protein of the predicted size following induction, demonstrating the use of the recombination cloning system. PCR amplicons of each ORF used in the cloning reactions were spotted onto glass slides to produce DNA microarrays representing 2035 genes of the gonococcal genome. Pilot experiments indicate that these arrays are suitable for the analysis of global gene expression in gonococci.

**Conclusion:**

This archived set of Gateway^® ^entry clones will facilitate high-throughput genomic and proteomic studies of gonococcal genes using a variety of expression and analysis systems. In addition, the DNA arrays produced will allow us to generate gene expression profiles of gonococci grown in a wide variety of conditions. Together, the resources produced in this work will facilitate experiments to dissect the molecular mechanisms of gonococcal pathogenesis on a global scale, and ultimately lead to the determination of the functions of unknown genes in the genome.

## Background

*Neisseria gonorrhoeae *(gonococcus), a Gram-negative diplococcus, is one of two pathogenic members of the Neisseriaceae family of bacteria. *N. gonorrhoeae *is the causative agent of the sexually transmitted disease, gonorrhea, one of the oldest documented infectious diseases. Gonorrheal disease has significant morbidity both in the US and worldwide. According to the Centers for Disease Control [1], >350,000 cases of gonorrhea were reported in the United States in 2002. The World Health Organization [2] estimates that over 19 million cases occur annually in the African continent alone. Treatment of gonorrhea is increasingly problematic due to the high frequency of acquisition of resistance to multiple antibiotics [3,4] and to the observation that gonococcal infection does not elicit protective immunity [5]. Gonorrheal infections, though not usually life-threatening, also enhance the transmission of HIV [6].

*N. gonorrhoeae *is strictly a human pathogen, with no known animal reservoir. The bacterium has no environmental niche, and cannot survive outside the human host. In adults, *N. gonorrhoeae *is acquired primarily through sexual contact. However, the eyes of newborn infants may be infected by passing through an infected birth canal, resulting in the condition, ophthalmia neonatorum, which can lead to blindness. In most cases, gonococcal infections are limited to the urogenital tract, causing urethritis in men and cervicitis in women. Occasionally, gonococci cross the epithelial barrier to enter the bloodstream causing septicemia, and transit to the joints resulting in arthritis. In women, ascending infections from the endocervix can result in pelvic inflammatory disease, salpingitis, tubal blockage and infertility. *N. gonorrhoeae *can also establish a carrier state in which apparently healthy individuals harbor culturable and infectious bacteria [7]. Carriers are thought be important for disease dissemination. A recent study revealed that the gonococcal carriage rate in women was 6.7% in a major metropolitan area [8].

Due to the importance of *N. gonorrhoeae *to human health, much research effort has focussed on identifying virulence factors and elucidating the biochemical interactions of these factors with the host cell [9-11], with the goal of developing vaccines and alternative treatments. It is clear, however, that in order to fully understand the capabilities of this organism to cause disease and elude eradication, it will be necessary to ultimately determine the functions of a great deal more of the gene products encoded by the gonococcal genome. The recent genome sequencing makes possible a variety of genomic and proteomic studies of *N. gonorrhoeae*. To facilitate such studies, we have cloned into a bacteriophage lambda-based recombination cloning system (Gateway^® ^[12], Invitrogen, Carlsbad, CA) 1624 of the 2189 predicted ORFs from the genome of *N. gonorrhoeae *strain FA1090 [13], and 48 of the 61 ORFs of the gonococcal genetic island (GGI) of strain MS11 [14,15]. This clone-set allows the generation of transcriptional and translational fusions without the necessity of additional cloning and sequencing. Coupled to the construction of this clone set, DNA microarrays were generated by spotting the insert DNA onto glass slides. Preliminary experiments with the clone set and DNA arrays indicate that this system is suitable for studies of expression of genes from *N. gonorrhoeae *in heterologous systems as well as for the study of global gene expression in this organism.

## Results

### Design of oligonucleotide primers

The goal of this project was to create a plasmid library representing the annotated ORFs of *N. gonorrhoeae*. The Gateway^® ^Cloning System from Invitrogen [12] was selected for several reasons. First, Gateway^® ^uses a recombination-based cloning method which has the added benefit that once an archival clone is sequence-validated, subsequent recombinants (ie. into expression vectors) do not need to be sequenced. Second, the initial clones lack transcriptional machinery such that the cloned ORFs are not expressed, thus avoiding problems from lethality due to troublesome gene products. Third, there are several expression and epitope-tagging vector options for the subsequent study of proteins encoded by the cloned ORFs, allowing a variety of approaches to studying their functions. The high efficiency also lends itself to high throughput approaches that are suitable for automation.

A total of 2071 unique primer pairs were successfully designed for the 2189 annotated ORFs of the FA1090 genome [13] and 61 ORFs of the GGI [14,15]. These primers were gene specific, and their termini contain sequences for recombination cloning into the entry vector, pDONR221 (Invitrogen). All primers were designed such that the final recombination product yielded the native start codon at the 5' end of the gene, including the 206 of the 229 predicted ORFs with alternative (non-ATG) start codons. Since nearly half of the genes of this group (110/229) are annotated (that is encode putative proteins with significant similarity to proteins of known function), it is very possible that they are functional genes in the gonococcus and were thus included in the clone set design. Whether or not they encode functional proteins will ultimately depend on the results of future expression and mutation studies. The 23 ORFs of this subset not included were less than 400 bp in length and considered too small for the Gateway^® ^system (see below). For the 149 ORFs encoding predicted proteins with an amino-terminal signal sequence (as identified by PSORT [16] and SignalP [17]), sequences encoding the signal sequence were removed and an ATG start codon placed at the 5' end of the remaining coding sequence. This was done to reduce problems of expression of hydrophobic signal sequences and to facilitate future expression and targeting studies for such recombinant proteins.

The primer design strategy was iterative, starting with an annealing temperature range of 62°–72°C and primer length set to 18 nt. All ORFs were included in the first iteration, and those ORFs for which a primer pair was not selected were subjected to subsequent iterations. In each subsequent iteration the primer length parameter was increased by one, up to a maximum primer length of 34 nt. Primers larger than this were not designed in part due to cost and convenience of oligo synthesis in our 96-well format. Next, the annealing temperature range was expanded 5°C in both the positive and negative directions and each primer size from 18 to 34 nt was tried again. No further iterations were attempted after the annealing temperature range exceeded 47°–87°C. Failing to meet either of these criteria resulted in a primer pair not being designed for the ORF. There were a total of 177 ORFs for which no primers were designed (see Additional file 2), 174 from FA1090 and 3 from the GGI. Most of these ORFs (165/177 = 93%) were less than 500 bp in length, and were not included since the Gateway^® ^system is reportedly less efficient for cloning fragments of this small size.

To each of the gene specific primer sequences for the 5' ends of the ORFs was added a 21 nt sequence including a consensus ribosome binding site (Shine-Dalgarno sequence). To each of the gene specific primer sequences for the 3' ends of the ORFs was added 20 nt corresponding to the 3' end of the *attB2 *site necessary for recombination into pDONR221. These were the primary PCR primers, the sequences of which are available on request. A single pair of primers was then designed for a secondary amplification to generate gene specific products containing the *attB *sites for the recombinatory cloning step. The 5' secondary primer contained the 24 nt *attB1 *tail and the 21 nt sequence (Shine-Dalgarno) common to all of the primary 5' primer sequences. The 3' secondary primer contained the remaining 10 nt of the *attB2 *site and the 20 nt *attB2 *sequence common to all of the primary 3' primer sequences. The *attB *sequences were as recommended in the Gateway^® ^manual.

### PCR amplification of ORFs

The first round of PCR, using gene specific primers, included a total of 2071 different primer pairs. Plates 1 and 2 were organized as pilot reactions and contained primers designed to amplify products ranging from 143 bp to 3485 bp in length. Genomic DNA from *N. gonorrhoeae *strain FA1090 was used as a template for the reactions. Agarose gel analysis of the amplicons showed that 173 of the 192 reactions yielded a product of the expected size, an efficiency of 90%. Plates 3–21 were then arranged with increasing size of expected product, with plate 3 containing the smallest products and plate 21 the largest. Several primer pairs (from plates 1 and 2) were included in plates 3–21 as internal controls. Plate 22 was an additional control plate, with one half of the plate (rows A-D) duplicated on the other half (rows E-H). Plate 23 corresponded to the genes of the GGI present in strain MS11 [14,15], and MS11 genomic DNA was used as the template for this plate of reactions. 5 μl of each reaction from the first round of PCR of each plate were run on agarose gels and scored for production of a product and whether it was of the expected size. The results showed 89% of the reactions to produce a product of the correct size.

Following the primary amplification with gene specific primers, all products (in the original 96-well format) were diluted 1:100 and an aliquot subjected to a secondary amplification using a pair of primers corresponding to the sequences common to all of the amplicons, and including additional sequences to generate a complete *attB *site for the cloning reaction. Aliquots of the secondary amplification were then used directly in the recombination reaction to generate entry clones.

### Construction of the library

Secondary amplicons were inserted into pDONR221 by *in vitro *recombination between the *attB *sites introduced at the ends of the amplicons and the *attP *sequences of the vector, maintaining the 96-well format arrangement. Cloning reactions were then transformed into *E. coli *strain DH5α and a portion of the transformation mix plated on LB plates containing kanamycin. Individual transformants were screened by PCR to determine the presence of and size of the insert. For the first round of screening, four independent transformants from each reaction were screened, maintaining the original 96-well format. A product of 350 bp was observed if no insert was present, a positive clone was identified as having a product 350 bp larger than the size of the corresponding primary PCR product.

For the initial set of 2147 transformation reactions, clones corresponding to 1165 genes were identified in the first four transformants screened, an efficiency of 54%. This efficiency varied greatly with the predicted size of insert, the smallest inserts (plate 3) were 83% positive in the first four screened and the next largest inserts (plate 20) had 17% positive in the first four screened. Plate 21, which had the largest inserts, only yielded 3 transformants as positive after several rounds of screening. Additional transformants for those clones not identified in the first round of screening were individually cultured, screened by PCR, and positive clones frozen down as they were identified. This approach yielded an additional 354 clones.

Following the initial rounds of screening, a list of missing clones was generated and the amplification and cloning steps repeated, optimizing several parameters and analyzing on an individual basis. This approach yielded an additional 283 clones, for a total of 1802 which were subsequently sequenced. Arrangement of the clones in plates for the master set and for sequencing was on an "as identified" basis, such that they are not arranged as in the original 96-well format. Each of the 21 plates contain viable clones in up to 95 of the 96 available positions, with position H12 (and additional wells on some plates) left empty for controls, providing a unique identity for several of the plates.

### Sequence verification of the clones

Transformants identified as having an insert of the predicted size were grown in 96-well plates and DNA isolated for sequence analysis. DNA isolation and sequencing was done by at the Human Genome Sequencing Center at Baylor College of Medicine (HGSC) using the same set of primers used to screen transformants for insert size. Sequence reads were posted onto an HGSC website and subsequently downloaded by FTP. DNA reads were processed initially using the STADEN DNA analysis software package [18]. Binary files were converted into .exp files using PREGAP4 to generate text files for each individual sequence read, with sequence quality cutoffs. These data are provided in Additional File 1 (AF1 exp sequence files.zip), and a description of the labelling scheme for the files is in the Methods section.

The data were next analyzed by BLAST [19] against the *N. gonorrhoeae *genome sequence database [13]. Clones expected to contain inserts from the GGI, not present in FA1090, were analyzed by BLAST 2 [20] using sequence of the individual GGI ORFs ([15], GenBank accession number AY803022). BLAST results were then manually tabulated in a file containing the expected gene for each archived clone. Of the 1802 sequenced clones, 58 were expected to be duplicates, leaving 1744 unique clones expected in the clone set. 1550 of the sequences were readable and corresponded to a predicted ORF from *N. gonorrhoeae*, 1399 of them unique, corresponding to 151 duplicates. Some duplicates were expected, and the remainder likely due to cross contamination from neighboring wells. Of those sequence validated, 55 were not in positions predicted. Most of these were due to human error, such as obvious well transpositions and numbering transpositions. 26 of these, however, had inserts in a backwards orientation and were incomplete. These clones will not be usable in subsequent recombination reactions using the Gateway^® ^system. Together, these data indicate that at present we have a collection of 1672 individual clones from *N. gonorrhoeae*, 48 of which are from the GGI, and 83% of which have been sequence validated. A list of genes in the clone set with sequencing result information can be found in Additional File 2 (AF2 NG clone set seq status.xls).

### Overexpression of randomly chosen ORFs

**Figure 1:**
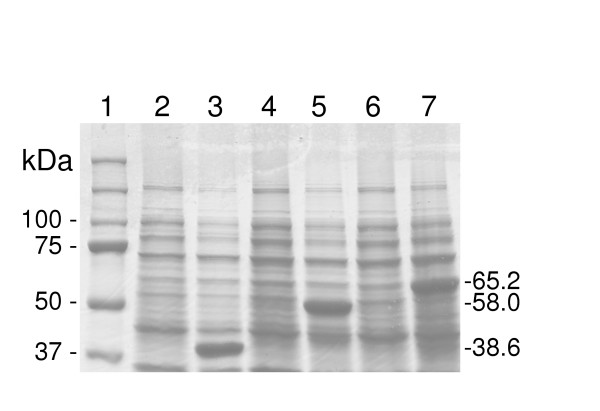
SDS-PAGE analysis of NG ORF-GST fusions. Equivalent amounts of total protein was boiled in sample buffer and electrophoresed on 10% polyacrylamide gels. Gels were stained with Coomassie Blue. Lane 1: MW markers; lane 2: NG1490(AspS)-GST, uninduced; lane 3: NG1490 induced (38.6 kdal); lane 4: NG1561(XthA)-GST uninduced; lane 5: NG1561 induced (58.0 kdal); lane 6: NG1641(PivNG)-GST uninduced; lane 7: NG1641 induced (65.2 kdal).

In order to examine the flexibility of using the clone set to construct various derivatives for which the Gateway^® ^system was designed, three randomly selected pDONR221 derivatives were used to create inducible glutathione-S-transferase (GST) fusions. Plasmids containing ORFs NG1490 (*aspS*, encodes aspartyl-tRNA synthetase), NG1561 (*xthA*, encodes exodeoxyribonuclease III), and NG1641 (*pivNG*, encodes a pilin gene inverting homolog, PivNG) are predicted to encode native proteins of 9.6, 29.0, and 36.2 kdal respectively. Plasmid DNA of pDONR221 derivatives containing these three genes were recombined with the destination vector pDEST15, an N-terminal GST fusion vector. The resulting recombinants were then transformed into *E. coli *BL21-AI for expression analysis. SDS-PAGE analysis of the proteins after a 2 hr induction with 0.2% L-arabinose is shown in Figure 1. The results show high levels of induction for each of the fusion proteins, with the sizes as predicted (GST adds 29 kdal).

### DNA microarray production

Since a very small portion of the secondary amplicons were used for the cloning reactions (5 μl of a 50 μl reaction), the remaining products were used to produce a set of DNA arrays for gene expression analysis. Gel electrophoresis (data not shown) indicated that the efficiency after the secondary amplification was 81%, representing 1681 ORFs of a total of 2071. To generate a more complete DNA array, a second set of primers (360) were designed to amplify internal portions of those ORFs not visible following the secondary PCR. Primers to amplify sequences of two small RNAs were also designed: NG0892.1, *ffs*, encodes the 4.5S RNA component of the gonococcal signal recognition particle [21]; and NG0880.1, *tmRNA*, encodes an RNA that tags abnormal proteins in the cell arising from stalled ribosomes and targets them for proteolysis [22]. Gel analysis of the amplicons produced from the internal PCR primers showed 294 of the 362 to produce products of the expected size, for an efficiency of 82%. Together, the cloning amplicons (1681) and internal ORF and RNA amplicons (294) represent a minimum of 1975 ORFs of the *N. gonorrhoeae *genome. This is a minimal estimate since all products (regardles of gel result) were to be spotted, and some products might be present, but at amounts too low to be visualized. DNA samples were processed and spotted onto glass slides as described in Methods. As a first test, the arrays were hybridized with a Cy3-labelled random nonamer oligonucleotide (Qiagen). A scan of the slide at 532 nm showed spots at the appropriate positions where DNA had been spotted, and blank spots at the buffer control spots. The next test was to hybridize the arrays with labelled genomic DNA. Total DNA from *N. gonorrhoeae *strain MS11 was digested with *Rsa*I and labelled by including Cy3-dCTP in a random-primed Klenow DNA polymerase reaction (Roche Applied Science, Indianapolis, IN). DNA arrays were hybridized and then scanned at 532 nm. Valid hybridization signals (at least one standard deviation above background) were detected for 98% of the spots expected to contain DNA, with those of the MS11-specific island comparable in intensity to those of FA1090 amplified DNA. Overall, the results showed valid signals for 2035 individual genes, with several in duplicate. The fact that this number is higher than expected based on agarose gel analysis of the amplicons before spotting (see above) indicates that some of the reactions produced a product, but at amounts too low to be visualized by ethidium bromide (EtBr) staining. Thus, these arrays represent 90% (2035/2250) of the predicted ORFs of *N. gonorrhoeae *(including 58 ORFs of the GGI; [14,15]) and 98% of those for which primers were designed.

## Discussion

Despite the advent of antibiotics in the 1940's, disease due to infection with *N. gonorrhoeae *remains a major health problem worldwide. The reasons for this are multi-fold. First, resistance to antibiotics by *N. gonorrhoeae *continues to rise [1,3,4]. In addition, treatment with high levels of broad spectrum antibiotics (which is frequently done since patients often do not return for follow up treatment) kills many bacteria of the (often beneficial) normal flora as well as the disease-causing microbe. Second, there is an incredibly high frequency of asymptomatic gonococcal infection, occurring in 5–10% of infected men and up to 50% of infected women. This represents a major reservoir for transmission of the infection. Furthermore, undiagnosed and untreated gonococcal salpingitis can lead to fallopian tube blockage. Partial blockage can result in ectopic pregnancy, which can be life threatening, and complete blockage of the fallopian tubes often leads to infertility. Third, development of a vaccine to protect against gonorrhea has been seriously hampered by the observation that gonococcal infection does not elicit protective immunity [5]. Patients can be reinfected following treatment, and can even be infected by multiple strains at a given time. Thus, alternative treatments and preventative strategies for gonococcal infection are sorely needed.

As a first step in the identification of such alternative treatments and preventatives, it will be necessary to more thoroughly understand the biology of the gonococcus and the molecular mechanisms involved in its interactions with the host environment. Much of the studies to date have focussed primarily on identifying the molecules on the surface of the bacterium that directly interact with the host, and the toxic moieties involved in damage to host cells. Many of the molecules identified are outer membrane (OM) components [23-27], and iron utilization proteins (reviewed in [28]). A few are secreted [29], or shed in blebs [30-32]. There has also been significant work in the identification of eukaryotic host cell receptors for gonococcal surface proteins (reviewed in [10]).

Interactions between gonococci and epithelial cells are beginning to be unraveled. Chen and Clark showed that contact with Hec-1-B human endocervical epithelial cells increases gonococcal infectiveness and that the process involves, in part, *de novo *protein synthesis by the bacterium [33]. The gonococcal type IV pilus (Tfp) and Opa proteins promote attachment, invasion and trans-epithelial trafficking. The mechanisms underlying Tfp- and Opa-mediated virulence are not yet understood, but these surface structures modulate a series of events in the infected epithelial cell, among them Ca^2+ ^fluxes [34,35], cortical rearrangements [36], and receptor phosphorylation [37-39]. Tfp retraction enhances the activation of stress-responsive kinases and the transcription of cytoprotective genes in the infected cell [40], and triggers the infected cell to produce a molecule that alters bacterial motility behavior [41]. Finally, binding of gonococci to primary urethral cells up-regulates anti-apoptotic factors [42]. These and other observations indicate that gonococcal infection requires the active participation of both the bacterium and the host cell.

The recent completion of the annotated genome sequence of *N. gonorrhoeae *[13,43], coupled with the development of high throughput methods for the analysis of gene expression and function, provide an opportunity to significantly advance the study of gonococcal biology and pathogenesis. Like many sequenced genomes, nearly half (44%) of the genes of the annotated gonococcal genome encode hypothetical proteins of unknown function. Furthermore, many of the annotations are based on homologies at the nucleotide and/or amino acid level, and the actual function of the gonococcal proteins have not been demonstrated. In order to realize the full potential of information gleaned from the genome sequence of this (and any) organism, it will be necessary to assign functions to all of the genes of the genome. The newly emerging fields of functional genomics and proteomics offer much promise towards achieving the goal of eventual assignment of functions for each and every gene in a given organism.

In this work, we describe the initial phases of the construction of an expression-capable clone set representing the annotated ORFs of the gonococcal genome using a recombination-based cloning system. The advantages of the system used for this set are numerous. 1) The original sequences in the clone set contain only the ORFs, not the gene expression sequences, thus avoiding the issue of expression-related lethality of the recombinants. 2) The clones can be transferred to a number of expression systems (prokaryotic and eukaryotic), allowing the regulation of genes for overproduction of proteins, or the production of proteins out of the context of the particular environments so as to study their functions. 3) The clones can also be transferred to vectors that result in epitope fusions, such as hexa-histidine, GST, green fluorescent protein (GFP), Lumio™, etc., to the proteins of interest. Protein fusions are useful in localizing proteins (within the bacterium or infected cell), in determining protein-interacting partners, in allowing smaller step purification protocols for structural and activity studies, and for antibody production. This ability has been demonstrated by constructing IPTG-inducible GST-fusions from three random clones from this set (Fig. 1). 4) The clone set is also catalogued in such a way that individual clones of interest are easily identified and recovered from the clone bank (see Additional file 2). 5) Entry clones can also be used to create knockouts by *in vitro *transposition [44,45] or shuttle mutagenesis [46] followed by transformation into naturally competent gonococci [47]. This system is also ammenable to automation, thus increasing the potential output and consistency in the data obtained.

The *N. gonorrhoeae *clone set thus far includes 1672 of the 2250 predicted ORFs of the genome [13], of which 83% are sequence-validated. Included in this set are 48 of the 61 ORFs of the MS11 GGI [14,15]. While this clone set is not yet complete, we believe these initial efforts have resulted in generating a valuable resource for the *Neisseria *research community. It is hoped that others in the community will share compatible reagents and add to the clone set, making it more comprehensive over time.

Coupled to the clone set construction, a PCR-amplicon based DNA microarray was generated. DNA microarrays are a powerful tool that allow one to measure relative transcript levels for essentially each gene of the genome simultaneously. These DNA arrays represent 2035 ORFs of the *N. gonorrhoeae *genome: 1977 from strain FA1090 [48] and 58 from the MS11 GGI [14], comprising 90% of the genes of the genome. Preliminary studies show that these arrays are suitable for examining global gene expression in *N. gonorrhoeae*.

Many bacterial pathogens are known to respond to changes in their physical environment, often integrating responses to several environmental signals via complex regulatory networks to control expression of a variety of genes [49-53]. The examples of regulatory systems characterized in gonococci are few, with the best characterized being the response to iron availability [54,55] and antimicrobial compounds [56,57]. The advent of microarray technologies has opened avenues of research on global gene expression in both prokaryotes and eukaryotes, providing opportunities for studying a variety of organisms, including such genetically intractable microbes as *Trepanema pallidum *[58] and *Chlamydia trachomatis *[59]. Thus, the use of DNA arrays will allow us to more fully explore the response of *N. gonorrhoeae *to environmental signals at the gene expression level. Since genes are typically only transcribed when the gene product function is required, expression profiles and cluster analyses will allow us to begin to determine the functions of unknown genes in the genome. Thus far, use of these DNA arrays has led to the identification of a regulator involved in the modulation of gonococcal gene expression upon adherence to epithelial cells in [45].

In summary, the tools described in this work represent a resource which will facilitate experiments to dissect the molecular mechanisms of gonococcal pathogenesis on a global scale. Combining these tools with the gonococcal infection models (tissue culture [60], organ culture [61-63], and the mouse model [64]), will allow us to make significant advances in the study of this important pathogen, thus providing us with the knowledge necessary to design therapeutics with which to treat and prevent gonococcal disease.

## Methods

### Gene predictions and initial sequence preparation

All ORF IDs for strain FA1090 reference records can be found at the STDGEN *Neisseria gonorrhoeae *annotated genome sequence database [13]. All gene sequences were prepared to include the natural start and stop codons. NG0540 and NG0634 have internal stop codons in the sequence database, and nothing was done to correct for this. Sequences of the ORFs of the MS11 GGI [15] have been deposited in the GenBank database with the accession number AY803022.

### Signal peptide identification

In order to determine whether the ORFs contained signal sequences, the programs PSORT [16] and SignalP [17] were employed. If both programs predicted a cleavable signal peptide for a given ORF sequence, that constituted "high support". The result of the signal peptide analyses showed 149 sequences with high support for a signal peptide. For those gene nucleotide sequences with a high support signal peptide, the nucleotide sequence representing the signal peptide was removed prior to cloning primer design. When both programs predicted different cleavage sites, the cleavage site that represented the shorter signal peptide was chosen. The rationale for this choice was that it would be better to include a bit of the signal peptide in the PCR product than to exclude a bit of the mature protein in the PCR product.

### Primer design

In the first cycle, the forward and reverse primers were fixed at the same length. This was due to the ease at which Primer3 [65] could be used. The input file was all gene sequences for which no signal peptide sequence was detected (see above). For the second cycle, the prim.aux file was manually inspected. This file contained information about successful primer picks where only a left or right primer could be picked. The strategy was to combine primers of different length. The potential for primer-dimer formation using this strategy was also assessed.

### *N.g. *ORF cloning

Primers for each of the genes were purchased from Illumina, Inc (San Diego, CA) and were designed to add sequences corresponding to part of the *attB *site necessary for recombination into the Gateway^® ^entry vector, pDONR221 (Invitrogen). Primary amplification was done using genomic DNA at a concentration of ~10 ng/reaction and gene specific primers at 2.5 μM. Reaction mix contained dNTPs, reaction buffer, MgCl_2 _and Taq polymerase as recommended by the manufacturer (Roche). Reactions conditions for primary PCR were as follows: denaturation at 94°C for 10 min; 10 cycles of 94°C 30 sec, 50°C 1 min, 74°C 1–5 min (depending on length of predicted product); 20 cycles of 94°C 30 sec, 55°C 1 min, 74°C 1–5 min (depending on length of predicted product); and a final extension at 74°C for 10 min. Following the primary amplification with the primer set, products were diluted 1:100 and 1 μl used as template for a secondary amplification using a pair of primers corresponding to the partial *attB *site common to all of the amplicons, and including additional sequences to generate a complete *attB *site for the cloning reaction. Reactions conditions for secondary PCR were as follows: denaturation at 94°C for 1 min; 5 cycles of 94°C 15 sec, 45°C 30 sec, 68°C 2 min; 15 cycles of 94°C 15 sec, 55°C 30 sec, 68°C 2 min. 5 μl of the 50 μl PCR was removed and used for cloning, and the remainder used for agarose gel analysis and printing of DNA arrays (N.g.array version 1.0, see below). Cloning reactions were performed according to the manufacturer's instructions (Invitrogen), transformed into *E. coli *strain DH5α, and transformants selected for kanamycin resistance. Individual transformants were picked in to wells of 96-well plates containing 100 μl L broth containing kanamycin (50 mg/l) and the same toothpick then used to place a small amount of bacteria directly into another plate containing a PCR cocktail. PCR was done using the M13 universal primers, which flank the *att *sites of the entry vector, pDONR221. A product of 350 bp was observed if no insert was present, providing an internal control for the PCR reactions. Individual clones were identified, stocked in duplicate, and grown for DNA isolation for sequencing.

### Sequencing

Sequencing runs from each end of the insert of each of the clones was determined to verify the ORF inserted. Complete sequence verification, (ie. both strands completely across the insert) was not done as it was determined to be impractical. DNA sequencing reactions were performed at the Baylor College of Medicine HGSC. Additional File 1 is a zipped file containing each of the sequence reads as .exp files generated using PREGAP4, and can be opened and read using word processing software. The file is separated into folders labelled SeqPlate #, which refers to sequencing plate number (1–21), and corresponds to the SP# designation in the list of clones in Additional File 2. SP18 sequencing reactions were done twice (SeqPlate 18-1, SeqPlate 18-2) as a sequencing reaction control, and the reactions of SP5 were analyzed three times (SeqPlate 5-1, SeqPlate 5-2, SeqPlate 5-3) as controls. SP20 does not exist, SP22 was not sent for sequencing, and the quality of SP17 sequence was too poor to be readable. The naming of the individual read files is as follows: BGACA(project code) # (1, 2, or 3 = reaction) D or F (primer D = forward, F = reverse) # (box, not same as SP) # (01–96; well, 1 = A1, 2 = B1, 9 = A2, and so on) A or B (run). For example: BGACA3D1701A (found in SeqPlate 1 file) corresponds to SP1 well A1 sequenced with the forward primer (5' end of ORF), result from the third reaction and the first gel.

### Gene expression analysis

pDONR221 derivatives chosen from the clone set were recombined with the destination vector, pDEST15 (Invitrogen), an N-terminal GST fusion vector. Plasmid DNA from the pDONR221 derivatives was isolated and incubated with pDEST15 in a LR recombination reaction performed according to the manufacturer's instructions. The recombination mixes were transformed into *E. coli *DH5α and transformants were selected on LB plates containing 50 mg/l Carbenicillin (Cb^50^). To test the possibility of false positive clones, the overexpression clones were tested for growth on chloramphenicol (20 mg/l), on which expression recombinants should not grow since the chloramphenicol resistance gene of pDEST15 is replaced by the insert. The resulting plasmids were purified and transformed into *E. coli *BL21-AI (Invitrogen) which expresses T7 RNA polymerase from the *araBAD *promotor, and transformants were selected on LB Cb^50 ^plates. BL21-AI strains containing the pDEST15 derivatives were grown overnight and used to inoculate fresh LB medium containing Cb^50 ^to an OD_600 _of 0.05. Expression of the GST-fusion proteins in *E. coli *BL21-AI was induced at an OD_600 _of 0.5 by addition of L-arabinose to a final concentration of 0.2%. Aliquots were removed after 2 hr and total proteins electropheresed on 10% polyacrylamide SDS gels [66].

### DNA microarray construction

PCR amplicons remaining from the cloning reactions (described above) were concentrated and then spotted in duplicate onto TeleChem SuperAmine glass slides (TeleChem International, Inc., Sunnyvale, CA) using a GeneMachines Omnigrid 100 (GeneMachines, Inc., San Carlos, CA) with 16 TeleChem Chipmaker 3 pins at the Genome Technology Support Facility (GTSF) at Michigan State University. Preparation of probes and hybridization conditions were as described [45]. Hybridized microarray slides were scanned using a GenePix 4000B scanner (Axon Instruments, Union City, CA) and images were processed and analyzed using GenePix version 4.1 software.

### Availability of clone set

The master library was used to generate a limited set of stock plates, which are stored at various locations as reference stocks. An additional set is stored at Michigan State University and is regarded as the working copy, which is the sole source of material for distribution. Interested parties should contact Cindy Arvidson (corresponding author) to arrange for a copy of the clone set. The clones will only be available as an intact set, individual clones or groups of clones are not available.

### Availability of microarrays

DNA arrays will be generated by re-amplification of the amplicons used for the recombination-cloning reactions using primers recognizing the common sequences at the ends of the amplicons at the MSU GTSF. Interested parties should contact Cindy Arvidson (corresponding author) to request slides, which will be produced upon request.

## Authors' contributions

Thomas Brettin: design of clone set construction, primer design

Michael R. Altherr: design of clone set construction, design and supervision of high-throughput PCR amplification of ORFs and recombination cloning into entry vectors

Ying Du: construction and tests of DNA microarrays, amplification and cloning of several ORFs not obtained in the initial batch cloning

Roxie M. Mason: cataloging of all cloning reactions processed at MSU (Arvidson lab), screening of transformants and preparation of clones for sequencing

Alexandra Friedrich: recombination of selected clones into expression vectors and analysis of proteins produced (Figure 1)

Laura Potter: initial project design, screening and cataloging of all cloning reactions processed at OHSU (So lab), screening of transformants and preparation of clones for sequencing

Chris Langford: batch BLAST analysis of sequencing results

Thomas J. Keller: batch BLAST analysis of sequencing results, wrote new program to faciliate analysis of sequence data

Jason Jens: processing of sequence data, batch program design for analysis of subset of clones from Gonococcal Genetic Island

Heather Howie: screening of transformants and preparation of clones for sequencing

Nathan J. Weyand: screening of transformants and preparation of clones for sequencing

Susan Clary: screening of transformants and preparation of clones for sequencing, amplification and cloning of several ORFs not obtained in the initial batch cloning

Kimberly Prichard: primer design

Susi Wachocki: high-throughput PCR amplification of ORFs and cloning into entry vectors

Erica Sodergren: sequencing of clone set

Joseph P. Dillard: contributed data of the Gonococcal Genetic Island prior to publication, participated in project design

George Weinstock: initial project design

Magdalene So: initial project design, supervison of all work performed at OHSU, substantial writing of manuscript

Cindy Grove Arvidson: initial project design, supervison of all work performed at MSU, screening of transformants from LANL, cloning of several ORFs not cloned in initial high-throughput cloning, collated and organanized all sequence data, primary writing of manuscript, corresponding author

## Supplementary Material

Additional File 2 Microsoft Excel file. Sheet 1: SP compiled – lists all clones sequenced and the results of the sequence analysis with respect to the gene expected and the gene identified by BLAST analysis of the sequence read. Sheet 2: entire ORF set – complete list of ORFs as annotated at [13] the status of each with respect to cloning primers designed, clone identified, and sequence verification. Click here for file

Additional File 1 Zipped file which includes all of the sequencing data as .exp files generated from binary data using PREGAP4. These .exp files contain all of the sequences with quality cutoffs included and can be opened with word processing programs. Click here for file
