# Supplementary material for: Expression capable library for studies of Neisseria gonorrhoeae, version 1.0
Source: BMC Microbiol. 2005 Sep 1;5:50. doi: 10.1186/1471-2180-5-50 (PMC1236931; doi:10.1186/1471-2180-5-50)
Supplement: Additional File 1 — Zipped file which includes all of the sequencing data as .exp files generated from binary data using PREGAP4. These .exp files contain all of the sequences with quality cutoffs included and can be opened with word processing programs. [file 1471-2180-5-50-S1.zip › Ng array ms revised 5-05.doc]

**Expression Capable Library for Studies of *Neisseria gonorrhoeae*, version 1.0**

Thomas Brettin^1^, Michael R. Altherr^1^, Ying Du^2^, Roxie M. Mason^2^, Alexandra Friedrich^3^, Laura Potter^3, 6^, Chris Langford^3^, Thomas J. Keller^3^, Jason Jens^2^, Heather Howie^3^, Nathan J. Weyand^3^, Susan Clary^3^, Kimberly Prichard^1^, Susi Wachocki^1^, Erica Sodergren^4^, Joseph P. Dillard^5^, George Weinstock^4^, Magdalene So^3^, and Cindy Grove Arvidson^2, 7^

running title: *N. gonorrhoeae* clone library

key words: *Neisseria gonorrhoeae*, expression library, DNA array

^1^Los Alamos National Laboratory, Los Alamos, NM; ^2^Department of Microbiology and Molecular Genetics, Michigan State University; East Lansing, MI; ^3^Department of Molecular Microbiology and Immunology, Oregon Health and Science University, Portland, OR; ^4^Human Genome Sequencing Center, Baylor College of Medicine, Houston TX; ^5^Department of Medical Microbiology and Immunology, University of Wisconsin Medical School, Madison, WI;

^6^Present address: Leicester Warwick Medical School, University of Warwick, Coventry, UK

^7^Corresponding author: phone: 517-355-6463 ext. 1573; FAX: 517-353-8957

e-mail addresses: TB, brettin@lanl.gov; MRA, altherr@lanl.gov; YD, duying@msu.edu; RMM, nelsonr6@msu.edu; AF, friedric@ohsu.edu; LP, l.c.potter@warwick.ac.uk; CL, langford@ohsu.edu; TJK, kellert@ohsu.edu; JJ, jensjaso@msu.edu; HH, howieh@ohsu.edu; NJW, weyandn@ohsu.edu; SC, clarys@ohsu.edu; KP, kjp@lanl.gov; SW, swachocki@direcway.com; ES, ericas@bcm.tmc.edu; JPD, jpdillard@facstaff.wisc.edu; GW, gwstock@bcm.tmc.edu; MS, somaggie@ohsu.edu; CGA, arvidso3@msu.edu

**ABSTRACT**

**Background:** The sexually transmitted disease, gonorrhea, is a serious health problem in developed as well as in developing countries, for which treatment continues to be a challenge. The recent completion of the annotated genome sequence of the causative agent, *Neisseria gonorrhoeae*, opens up an entirely new set of approaches for studying this organism and the diseases it causes. Here, we describe the initial phases of the construction of an expression-capable clone set representing the annotated protein-coding ORFs of the gonococcal genome using a recombination-based cloning system.

**Results:** The clone set thus far includes 1671 of the 2250 predicted ORFs of the *N. gonorrhoeae* genome, of which 84% are sequence-validated. Included in this set are 48 of the 61 ORFs of the gonococcal genetic island of strain MS11, not present in the sequenced genome of strain FA1090. L-arabinose-inducible glutathione-S-transferase (GST)-fusions were constructed from random clones and each was shown to express a fusion protein of the predicted size following induction, demonstrating the use of the recombination cloning system. Coupled to the clone set construction, DNA microarrays were produced. PCR amplicons of each ORF used in the cloning reactions were spotted onto glass slides, and the arrays shown to represent 2035 genes of the gonococcal genome. Gene expression analysis comparing expression in otherwise isogenic piliated (P^+^) and non-piliated (P^-^) strains of *N. gonorrhoeae* showed the most highly differentially expressed gene to be *pilE*, which encodes pilin, demonstrating the utility of these arrays for the analysis of global gene expression in gonococci.

**Conclusions:** The archived set of Gateway® entry clones will facilitate high-throughput genomic and proteomic studies of gonococcal genes using a variety of expression and analysis systems. In addition, the DNA arrays produced will allow us to generate gene expression profiles of gonococci grown in a wide variety of conditions, and ultimately lead to the determination of the functions of many of the unknown genes in the genome. Together, the resources produced in this work will facilitate experiments to dissect the molecular mechanisms of gonococcal pathogenesis on a global scale.

**BACKGROUND**

*Neisseria gonorrhoeae* (GC, gonococcus), a Gram-negative diplococcus, is one of two pathogenic members of the Neisseriaceae family of bacteria. *N. gonorrhoeae* is the causative agent of the sexually transmitted disease, gonorrhea, one of the oldest documented infectious diseases. Gonorrheal disease has significant morbidity both in the US and worldwide. According to the Centers for Disease Control [1], >350,000 cases of gonorrhea were reported in the United States in 2002. The World Health Organization [2] estimates that over 19 million cases occur annually in the African continent alone. Treatment of gonorrhea is increasingly problematic due to the high frequency of acquisition of resistance to multiple antibiotics [3, 4] and to the observation that gonococcal infection does not elicit protective immunity [5]. Gonorrheal infections, though not usually life-threatening, also enhance the transmission of HIV [6].

*N. gonorrhoeae* is strictly a human pathogen, with no known animal reservoir. The bacterium has no environmental niche, and cannot survive outside the human host. In adults, *N. gonorrhoeae* is acquired primarily through sexual contact. However, the eyes of newborn infants may be infected by passing through an infected birth canal, resulting in the condition, ophthalmia neonatorum, which can lead to blindness. In most cases, gonococcal infections are limited to the urogenital tract, causing urethritis in men and cervicitis in women. Occasionally, gonococci cross the epithelial barrier to enter the bloodstream causing septicemia, and transit to the joints resulting in arthritis. In women, ascending infections from the endocervix can result in pelvic inflammatory disease, salpingitis, tubal blockage and infertility. *N. gonorrhoeae* can also establish a carrier state in which apparently healthy individuals harbor culturable and infectious bacteria [7]. Carriers are thought be important for disease dissemination. A recent study revealed that the gonococcal carriage rate in women was 6.7% in a major metropolitan area [8].

Due to the importance of *N. gonorrhoeae* to human health, much research effort has focussed on identifying virulence factors and elucidating the biochemical interactions of these factors with the host cell [9, 10, 11], with the goal of developing vaccines and alternative treatments. It is clear, however, that in order to fully understand the capabilities of this organism to cause disease and elude eradication, it will be necessary to ultimately determine the functions of a great deal more of the gene products encoded by the gonococcal genome. In addition, the advent of gene chip, or microarray, technologies has opened avenues of research on global gene expression in both prokaryotes and eukaryotes, providing opportunities for studying a variety of organisms, including such genetically intractable microbes as *Trepanema pallidum* [12] and *Chlamydia trachomatis* [13].

Recent developments in genome sequencing make possible a variety of genomic and proteomic studies of *N. gonorrhoeae*. To facilitate such studies, we have cloned into a bacteriophage lambda-based recombination cloning system (Gateway®, Invitrogen, [14]) 1623 of the 2189 predicted ORFs from the genome of *N. gonorrhoeae* strain FA1090 [15], and 48 of the 61 ORFs of the gonococcal genetic island (GGI) of strain MS11 [16, 17]. This clone-set allows the generation of transcriptional and translational fusions without the necessity of additional cloning and sequencing. Coupled to the construction of this clone set, DNA microarrays were generated by PCR amplification of the insert DNA using a universal primer pair, followed by spotting onto glass slides. Preliminary experiments with the clone set and DNA arrays indicate that this system is suitable for studies of global gene expression in *N. gonorrhoeae* as well as expression of genes from *N. gonorrhoeae* in heterologous systems.

**RESULTS**

**Design of oligonucleotide primers.** The goal of this project was to create a plasmid library representing the annotated ORFs of *N. gonorrhoeae*. The Gateway® Cloning System from Invitrogen [14] was selected for several reasons.

First, Gateway uses a recombination-based cloning method, which is highly efficient, is of high fidelity, and has the added benefit that once an archival clone is sequence-validated, subsequent recombinants (ie. into expression vectors) do not need to be sequenced. Second, the initial clones lack transcriptional machinery such that the cloned ORFs are not expressed, thus avoiding problems from lethality due to troublesome gene products. Third, there are several expression and epitope-tagging vector options for the subsequent study of proteins encoded by the cloned ORFs, allowing a variety of approaches to studying their functions. The high efficiency also lends itself to high throughput approaches that are suitable for automation.

A total of 2071 unique primer pairs were successfully designed for the 2189 annotated ORFs of the FA1090 genome [15] and 61 ORFs of the GGI [16, 17]. These primers were gene specific, and their termini contain sequences for recombination cloning into the Gateway® entry vector, pDONR221 (Invitrogen).

The primer design strategy was iterative, starting with an annealing temperature range of 62°-72°C and primer length set to 18 nt. All ORFs were included in the first iteration, and those ORFs for which a primer pair was not selected were subjected to subsequent iterations. In each subsequent iteration the primer length parameter was increased by one, up to a maximum primer length of 34 nt. Primers larger than this were not designed in part due to cost and convenience of oligo synthesis in our 96-well format. Next, the annealing temperature range was expanded 5°C in both the positive and negative directions and each primer size from 18 to 34 nt was tried again. No further iterations were attempted after the annealing temperature range exceeded 47°-87°C. Failing to meet either of these criteria resulted in a primer pair not being designed for the ORF. There were a total of 177 ORFs for which no primers were designed, 174 from FA1090 and 3 from the GGI. Most of these ORFs (165/177 = 93%) were less than 500 bp in length, and were not included since the Gateway® system is reportedly less efficient for cloning fragments of this small size.

To each of the gene specific primer sequences for the 5’ ends of the ORFs was added a 21 nt sequence including a consensus ribosome binding site (Shine-Dalgarno sequence). To each of the gene specific primer sequences for the 3’ ends of the ORFs was added 20 nt corresponding to the 3’ end of the *attB2* site necessary for recombination into the Gateway® vectors. These were the primary PCR primers, the sequences of which are available on request. A single pair of primers was then designed for a secondary amplification to generate gene specific products containing the *attB* sites for the recombinatory cloning step. The 5’ secondary primer contained the 24 nt *attB1* tail and the 21 nt sequence (Shine-Dalgarno) common to all of the primary 5’ primer sequences. The 3’ secondary primer contained the remaining 10 nt of the *attB2* site and the 20 nt *attB2* sequence common to all of the primary 3’ primer sequences. The *attB* sequences were as recommended in the Gateway® manual.

**PCR amplification of ORFs.** The first round of PCR, using gene specific primers, included a total of 2071 different primer pairs. Plates 1 and 2 were organized as pilot reactions and contained primers designed to amplify products ranging from 143 bp to 3485 bp in length. Genomic DNA from *N. gonorrhoeae* strain FA1090 was used as a template for the reactions. Agarose gel analysis of the amplicons showed that 173 of the 192 reactions yielded a product of the expected size, an efficiency of 90%. Plates 3-21 were then arranged with increasing size of expected product, with plate 3 containing the smallest products and plate 21 the largest. Several primer pairs (from plates 1 and 2) were included in plates 3-21 as internal controls. Plate 22 was an additional control plate, with one half of the plate (rows A-D) duplicated on the other half (rows E-H). Plate 23 corresponded to the genes of the GGI present in strain MS11 [16, 17], and MS11 genomic DNA was used as the template for this plate of reactions. 5 μl of each reaction from the first round of PCR was run on agarose gels and scored for production of a product and whether it was of the expected size. The results showed 89% of the reactions to produce a product of the correct size.

Following the primary amplification with gene specific primers, all products (in the original 96-well format) were diluted 1:100 and an aliquot subjected to a secondary amplification using a pair of primers corresponding to the sequences common to all of the amplicons, and including additional sequences to generate a complete *attB* site for the cloning reaction. Aliquots of the secondary amplification were then used directly in the recombination reaction to generate entry clones.

**Construction of the library.** Secondary amplicons were inserted into the Gateway® entry vector pDONR221 by *in vitro* recombination between the *attB* sites introduced at the ends of the amplicons and the *attP* sequences of the vector, maintaining the 96-well format arrangement. Cloning reactions were then transformed into *E. coli* strain DH5α and a portion of the transformation mix plated on LB plates containing Kan. Individual transformants were screened by PCR to determine the presence of and size of the insert. For the first round of screening, four independent transformants from each reaction were screened, maintaining the original 96-well format. A product of 350 bp was observed if no insert was present, a positive clone was identified as having a product 350 bp larger than the size of the corresponding primary PCR product.

For the initial set of 2147 transformation reactions, clones corresponding to 1165 genes were identified in the first four transformants screened, an efficiency of 54%. This efficiency varied greatly with the predicted size of insert, the smallest inserts (plate 3) were 83% positive in the first four screened and the next largest inserts (plate 20) had 17% positive in the first four screened. Plate 21, which had the largest inserts, only yielded 3 transformants as positive after several rounds of screening. Additional transformants for those clones not identified in the first round of screening were picked and screened using a cherry-picking approach, with positives frozen down as they were identified. This approach yielded an additional 354 positive clones.

Following the initial rounds of screening, a list of missing clones was generated and the amplification and cloning steps repeated, optimizing several parameters and analyzing on an individual basis. This approach yielded an additional 283 clones, for a total of 1802 which were subsequently sequenced. Arrangement of the clones in plates for the master set and for sequencing was on an “as identified” basis, such that they are not arranged as in the original 96-well format. Each of the plates contain viable clones in up to 95 of the 96 available positions, with position H12 (and additional wells on some plates) left empty for controls, providing a unique identity for several of the plates.

**Sequence verification of the clones.** Transformants identified as having an insert of the predicted size were grown in 96-well plates and DNA isolated for sequence analysis. DNA isolation and sequencing was done by at the Human Genome Sequencing Center at Baylor College of Medicine (HGSC) using the same set of primers used to screen transformants for insert size. Sequence reads were posted onto an HGSC website and subsequently downloaded by FTP. DNA reads were processed initially using the STADEN DNA analysis software package [18]. Binary files were converted into .exp files using PREGAP4 to generate text files for each individual sequence read, with sequence quality cutoffs. The data were next analyzed by BLAST [19] against the *N. gonorrhoeae* genome sequence database [15]. Clones expected to contain inserts from the GGI, not present in FA1090, were analyzed by BLAST 2 [20] using sequence of the individual GGI ORFs ([17], GenBank accession number AY803022). BLAST results were then manually tabulated in a file containing the expected gene for each archived clone. Of the 1802 sequenced clones, 58 were expected to be duplicates, leaving 1744 unique clones expected in the clone set. 1550 of the sequences were readable and corresponded to a predicted ORF from *N. gonorrhoeae*, 1399 of them unique, corresponding to 151 duplicates. Some duplicates were expected, and the remainder likely due to cross contamination from neighboring wells. Of those sequence validated, 55 were not in positions predicted. Most of these were due to human error, such as obvious well transpositions and numbering transpositions. 26 of these, however, had inserts in a backwards orientation and were incomplete. These clones will not be usable in subsequent recombination reactions using the Gateway® system. Together, these data indicate that at present we have a collection of 1671 individual clones from *N. gonorrhoeae*, 48 of which are from the GGI, and 84% of which have been sequence validated. Raw sequence data and/or BLAST results are available on request.

**First generation microarray production and analysis.** Since a very small portion of the secondary amplicons were used for the cloning reactions (5 μl of a 50 μl reaction), the remaining products were used to produce a set of DNA arrays for gene expression analysis. Gel electrophoresis (data not shown) indicated that the efficiency after the secondary amplification was 81%, representing 1681 ORFs of a total of 2071. To generate a more complete DNA array, a second set of primers (360) were designed to amplify internal portions of those ORFs not visible following the secondary PCR. Primers to amplify sequences of two small RNAs were also designed: NG0892.1, *ffs*, encodes the 4.5S RNA component of the gonococcal signal recognition particle [21]; and NG0880.1, *tmRNA*, encodes an RNA that tags abnormal proteins in the cell arising from stalled ribosomes and targets them for proteolysis [22]. Gel analysis of the amplicons produced from the internal PCR primers showed 294 of the 362 to produce products of the expected size, for an efficiency of 82%. Together, the cloning amplicons (1681) and internal ORF and RNA amplicons (294) represent a minimum of 1975 ORFs of the *N. gonorrhoeae* genome. This is a minimal estimate since all products (regardles of gel result) were to be spotted, and some products might be present, but at amounts too low to be visualized.

DNA samples were processed and spotted onto glass slides to produce a version 1.0 DNA array (see Materials and Methods). As a first test, the arrays were hybridized with a Cy3-labelled random nonamer oligonucleotide (Qiagen). A scan of the slide at 532 nm showed spots at the appropriate positions where DNA had been spotted, and blank spots at the buffer control spots.

The next test was to hybridize the arrays with labelled genomic DNA. Total DNA from *N. gonorrhoeae* strain MS11 was digested with *Rsa*I and labelled by including Cy3-dCTP in a random-primed Klenow DNA polymerase reaction (Roche Applied Science, Indianapolis, IN). DNA arrays were hybridized as described in Materials and Methods and then scanned at 532 nm and images processed and analyzed. Valid hybridization signals (at least one standard deviation above background) were detected for 98% of the spots expected to contain DNA, with those of the MS11-specific island comparable in intensity to those of FA1090 amplified DNA. Overall, the results showed valid signals for 2035 individual genes, with several in duplicate. The fact that this number is higher than expected based on agarose gel analysis of the amplicons before spotting (see above) indicates that some of the reactions produced a product, but at amounts too low to be visualized by ethidium bromide (EtBr) staining. Thus, these arrays represent 90% (2035/2250) of the predicted ORFs of *N. gonorrhoeae* (including 58 ORFs of the GGI; [16, 17]) and 98% of those for which primers were designed.

The arrays were next used to analyze global gene expression in *N. gonorrhoeae* strains differing only in their expression of an abundant gonococcal protein, pilin. Pilin is the major protein component of the gonococcal type IV pilus, a primary adhesin which mediates attachment of the bacterium to host cells [23] and is encoded by the *pilE* expression locus [24]. It has been shown that there are several proteins that differ in production and localization in piliated versus non-piliated gonococci [25]. However, the identity of the proteins and the mechanism(s) by which the differential production and localization occurs is not known.

*N. gonorrhoeae* strains MS11 and an otherwise isogenic derivative of MS11 containing large deletions in both *pilE1* and *pilE2*, MS11-307 [26] were grown in GC broth and RNA isolated. Labelled cDNA was synthesized and hybridized to array slides. Following appropriate washes, the slides were scanned at both 532 and 635 nm and images analyzed. Data were normalized to eliminate labelling artifacts (intensity based normalization) and the ratios of expression determined. Outliers were identified as genes with expression ratios (log_2_ transformed) greater than 2.5 standard deviations from the mean [27]. Data from 3 independent experiments (including a dye swap) were collated and average ratios determined for genes that were identified as an outlier in at least 2 of 3 experiments (Tables 1 and 2).

As expected, *pilE* (70-fold) and several *pilS* sequences (loci containing partial variant pilin genes which serve as a reservoir for recombination to yield antigenically variant pilin proteins, [28]) were among the highest differentially expressed, essentially expressed only in the P^+^ strain (MS11). There are two explanations for the observation that *pilS* loci (which are not complete ORFs, but were included in the arrays) produce a signal from RNA isolated from piliated gonococci. First, there is significant homology between different *pilS* sequences and the variable regions of the expressed pilin gene (*pilE*) which is necessary for the RecA-mediated recombination which leads to pilin antigenic variation [29, 30, 28]. Since our array slides are spotted with entire ORF PCR amplicons and not short oligonucleotides, we do not expect to be able to differentiate between various *pil* loci in our hybridizations. Second, recombination between *pilS* and *pilE* loci occurs at a very high frequency in gonococci [28, 31, 32]. The population of piliated bacteria from which RNA was isolated likely has more than one pilin variant being expressed, which would result in a mixture of *pilE* sequences being expressed, and hybridization to all of the *pilS* as well as *pilE* amplicons spotted on the array slide would be observed.

The next highest up-regulated in the wild-type (P^+^) strain were two genes, *bfrA* and *bfrB* (3.8-fold and 4.0-fold, respectively), which encode the two subunits of bacterioferritin, an iron storage protein that prevents the toxic effects of intracellular iron which can occur during aerobic growth. Few genes were identified as significantly up-regulated (≥ 2-fold) in P- bacteria (MS11-307, Table 2). Several genes, however, were up-regulated ~1.5-fold in the P- strain (Table 3). The expression ratios of these genes were slightly below the cutoff in our outlier analysis, yet were very consistent from between experiments. Interestingly, nearly all of the genes in this category encode proteins involved in gene expression, including *rpoA* (encodes RNA polymerase a subunit), *fusA* (encodes translation elongation factor G), and several ribosomal proteins. Since pilin is one of the more abundant proteins in wild-type (P^+^) gonococci, it is curious that a strain no longer producing pilin up-regulates the transcription-translation machinery. This correlates with the observation that P- gonococci grow faster than P^+^, and increased translation would be expected when growth rate is increased. There may be a significant saving of energy when gonococci are not producing pilin protein, assembling pili, and continually extending and retracting the pili, all of which require significant energy output [33, 34].

**Overexpression of randomly chosen ORFs.** In order to examine the flexibility of using the clone set to construct various derivatives for which the Gateway system was designed, three randomly selected pDONR221 derivatives were used to create inducible glutathione-S-transferase (GST) fusions. Plasmids containing ORFs NG1490 (*aspS*), NG1561 (*xthA*), and NG1641 (*pivNG*) are predicted to encode native proteins of 9.6, 29.0, and 36.2 kdal respectively.

Plasmid DNA of pDONR221 derivatives containing these three genes were recombined with the destination vector pDEST15, an N-terminal GST fusion vector. The resulting recombinants

were then transformed into *E. coli* BL21-AI for expression analysis. SDS-PAGE analysis of the proteins after a 2 hr induction with 0.2% L-arabinose is shown in Figure 2. The results show high levels of induction for each of the fusion proteins, with the sizes as predicted (GST adds 29 kdal).

**DISCUSSION**

Despite the advent of antibiotics in the 1940's, disease due to infection with *N. gonorrhoeae* remains a major health problem worldwide. The reasons for this are multi-fold. First, resistance to antibiotics by *N. gonorrhoeae* continues to rise [1, 3, 4]. In addition, treatment with high levels of broad spectrum antibiotics (which is frequently done since patients often do not return for follow up treatment) kills many bacteria of the (often beneficial) normal flora as well as the disease-causing microbe. Second, there is an incredibly high frequency of asymptomatic gonococcal infection, occurring in 5-10% of infected men and up to 50% of infected women. This represents a major reservoir for transmission of the infection. Furthermore, undiagnosed and untreated gonococcal salpingitis can lead to fallopian tube blockage. Partial blockage can result in ectopic pregnancy, which can be life threatening, and complete blockage of the fallopian tubes often leads to infertility. Third, development of a vaccine to protect against gonorrhea has been seriously hampered by the observation that gonococcal infection does not elicit protective immunity [5]. Patients can be reinfected following treatment, and can even be infected by multiple strains at a given time. Thus, alternative treatments and preventative strategies for gonococcal infection are sorely needed.

As a first step in the identification of such alternative treatments and preventatives, it will be necessary to more thoroughly understand the biology of the gonococcus and the molecular mechanisms involved in its interactions with the host environment. Much of the studies to date have focussed primarily on identifying the molecules on the surface of the bacterium that directly interact with the host, and the toxic moieties involved in damage to host cells. Many of the molecules identified are outer membrane (OM) components [35, 36, 37, 38, 39, 40, 41, 42, 43, 44, 45], and iron utilization proteins (reviewed in [46]). A few, such as the IgA1 protease, are secreted [47, 48], or shed in blebs [49, 50, 51, 52, 53, 54]. There has also been significant work in the identification of eukaryotic host cell receptors for gonococcal surface proteins [55, 56, 57, 58, 59, 60, 61, 62, 63], reviewed in [10].

Interactions between gonococci and epithelial cells are beginning to be unraveled. Chen and Clark showed that contact with Hec-1-B human endocervical epithelial cells increases gonococcal infectiveness and that the process involves, in part, *de novo* protein synthesis by the bacterium [64]. The gonococcal type IV pilus (Tfp) and Opa proteins promote attachment, invasion and trans-epithelial trafficking. The mechanisms underlying Tfp- and Opa-mediated virulence are not yet understood, but these surface structures modulate a series of events in the infected epithelial cell, among them, Ca^2+^ fluxes [65, 66, 67], cortical rearrangements [68], and receptor phosphorylation [69, 70, 71]. Tfp retraction enhances the activation of stress-responsive kinases and the transcription of cytoprotective genes in the infected cell (Lee et al., manuscript submitted; Howie et al., manuscript submitted). Tfp retraction also triggers the infected cell to produce the phospholipid PI[3,4,5]P3 which, in turn, alters bacterial motility behavior (Lee et al., manuscript submitted). Thus, like Enteropathogenic *E. coli* [72] gonococci induce the infected cell to produce a bacterial effector. Finally, binding of gonococci to primary urethral cells up-regulates anti-apoptotic factors [73]. These and other observations indicate that gonococcal infection requires the active participation of both the bacterium and the host cell.

The recent completion of the annotated genome sequence of *N. gonorrhoeae* [15, 74], coupled with the development of high throughput methods for the analysis of gene expression and function, provide an opportunity to significantly advance the study of gonococcal biology and pathogenesis. Like many sequenced genomes, nearly half (44%) of the genes of the annotated gonococcal genome encode hypothetical proteins of unknown function. Furthermore, many of the annotations are based on homologies at the nucleotide and/or amino acid level, and the actual function of the gonococcal proteins have not been demonstrated. In order to realize the full potential of information gleaned from the genome sequence of this (and any) organism, it will be necessary to assign functions to all of the genes of the genome. The newly emerging fields of functional genomics and proteomics offer much promise towards achieving the goal of eventual assignment of functions for each and every gene in a given organism.

In this work, we describe the initial phases of the construction of an expression-capable clone set representing the annotated ORFs of the gonococcal genome using a recombination-based cloning system. The advantages of the Gateway® vector system used for this set are numerous. 1) The original sequences in the clone set contain only the ORFs, not the gene expression sequences, thus avoiding the issue of expression-related lethality of the recombinants. 2) The clones can be transferred to a number of expression systems (prokaryotic and eukaryotic), allowing the regulation of genes for overproduction of proteins, or the production of proteins out of the context of the particular environments so as to study their functions. 3) The clones can also be transferred to vectors that result in epitope fusions, such as hexa-histidine, GST, green fluorescent protein (GFP), Lumio™, etc., to the proteins of interest. Protein fusions are useful in localizing proteins (within the bacterium or infected cell), in determining protein-interacting partners, in allowing smaller step purification protocols for structural and activity studies, and for antibody production. This ability has been demonstrated by constructing IPTG-inducible GST-fusions from three random clones from this set (Fig. 2). 4) The clone set is also catalogued in such a way that individual clones of interest are easily identified and recovered from the clone bank. 5) Entry clones can also be used to create knockouts by *in vitro* transposition [75] or shuttle mutagenesis [76] followed by transformation into naturally competent gonococci [77]. The ORFeome-wide approaches afforded by this system are ammenable to automation, thus increasing the potential output and consistency in the data obtained.

The *N. gonorrhoeae* clone set thus far includes 1671 of the 2250 predicted ORFs of the genome [15], of which 84% are sequence-validated. Included in this set are 48 of the 61 ORFs of the MS11 GGI [16, 17]. While this clone set is not yet complete, we believe these initial efforts have resulted in generating a valuable resource for the *Neisseria* research community. It is hoped that others in the community will share compatible reagents and add to the clone set, making it more comprehensive over time.

Coupled to the clone set construction, a PCR-amplicon based DNA microarray was generated. DNA microarrays are a powerful tool that allow one to measure relative transcript levels for essentially each gene of the genome simultaneously. These DNA arrays represent 2035 ORFs of the *N. gonorrhoeae* genome: 1977 from strain FA1090 [78] and 58 from the MS11 GGI [16], comprising 90% of the genes of the genome. Preliminary studies show that these arrays are suitable for examining global gene expression in *N. gonorrhoeae*. Gene expression in an otherwise isogenic pair of strains in which the only difference is the deletion of the pilin expression loci (*pilE1* and *pilE2*) in one of the strains, MS11-307 [26], was compared on a genome wide scale using these arrays. Our results (Tables 1 and 2) showed that the most highly differentially expressed genes were *pilE*, and the partial copies of pilin genes (*pilS*) found scattered over the gonococccal genome that are involved in the complex mechanism of pilin antigenic variation [32, 28].

Many bacterial pathogens are known to respond to changes in their physical environment, often integrating responses to several environmental signals via complex regulatory networks to control expression of a variety of genes [79, 80, 81, 82, 83, 84, 85]. The examples of regulatory systems characterized in gonococci are few, with the best characterized being the response to iron availability [86, 87] and antimicrobial compounds [88, 89]. Relatively little else is known about the response of *N. gonorrhoeae* to host cell contact and other environmental signals at the gene expression level. The use of our gene chips (N.g. version 1.0) will allow us to generate gene expression profiles of gonococci grown in a wide variety of conditions. Since genes are typically only transcribed when the gene product function is required, expression profiles and cluster analyses will allow us to begin to determine the functions of unknown genes in the genome. Gonococcal gene expression is likely to occur during infection, and this technology will allow us to monitor these changes on a global scale [90]. The development and use of DNA microarrays for the closely related *N. meningitidis* has already yielded significant information on gene expression in this bacterium in response to iron availability [91], heat-shock [92], and host-cell contact [93, 94].

The DNA arrays produced here will also allow us to begin to identify and characterize regulatory networks in *N. gonorrhoeae*. Gonococcal strains can be constructed in which regulatory genes (of the clone set) are insertionally inactivated [75] and/or expressed under a regulatable promoter [95]. The resulting strains can then be used to examine changes in regulatory patterns on a genome wide scale. Ultimately, we will be able to carefully dissect the regulatory systems involved, the specific signals involved, and the mechanisms by which the regulation occurs. Since there are few well-characterized virulence regulatory systems identified in the pathogenic Neisseriae, the results of these experiments will provide new and vital information in understanding how these pathogens respond to their environment and how this is related to the disease process. We have begun to make use of these arrays for host-pathogen interaction studies and have identified a putative regulatory system that mediates the response of gonococci to host cell contact (Y. Du, J. Lenz, and C. G. Arvidson, submitted).

The tools described in this work represent a resource which will facilitate experiments to dissect the molecular mechanisms of gonococcal pathogenesis on a global scale. Combining these tools with the gonococcal infection models (tissue culture [96], organ culture [97, 98, 99], and the mouse model [100]), will allow us to make significant advances in the study of this important pathogen, thus providing us with the knowledge necessary to design therapeutics with which to treat and prevent gonococcal disease.

**Availability of Materials**

**DNA arrays.** The version 1.0 array (as described in this work) is limited due to the amount of material available for printing and is not available for distribution. A version 2.0 array will be generated by re-amplification of the amplicons used for the version 1.0 arrays using primers recognizing the common sequences at the ends of the amplicons necessary for the recombination-cloning reactions at the MSU GTSF. Interested parties should contact Cindy Arvidson (corresponding author) to request slides, which will be produced upon request.

**Clone set.** The master library was used to generate a limited set of stock plates, which are stored at various locations as reference stocks. An additional set is stored at Michigan State University and is regarded as the working copy, which is the sole source of material for distribution. Interested parties should contact Cindy Arvidson (corresponding author) to arrange for a copy of the clone set. The clones will only be available as an intact set, individual clones or groups of clones are not available.

**MATERIALS AND METHODS**

**Gene predictions and initial sequence preparation.** All ORF IDs for strain FA1090 reference records can be found at the STDGEN *Neisseria gonorrhoeae* annotated genome sequence database [15]. All gene sequences were prepared to include the natural stop codon and and ATG start codon. NG0540 and NG0634 have internal stop codons in the sequence database, and nothing was done to correct for this. Sequences of the ORFs of the MS11 GGI [17] have been deposited in the GenBank database with the accession number AY803022.

**Signal peptide identification.** We started with 2050 gene nucleotide sequences that we believe to be a best approximation to the complete gene set in *N. gonorrhoeae* strain FA1090 [78]. In order to determine whether the ORFs contained signal sequences, the programs PSORT [101] and SignalP [102] were employed. If both programs predicted a cleavable signal peptide for a given ORF sequence, that constituted “high support”. The result of the signal peptide analyses showed 149 sequences with high support for a signal peptide. For those gene nucleotide sequences with a high support signal peptide, the nucleotide sequence representing the signal peptide was removed prior to cloning primer design. When both programs predicted different cleavage sites, the cleavage site that represented the shorter signal peptide was chosen. The rationale for this choice was that it would be better to include a bit of the signal peptide in the PCR product than to exclude a bit of the mature protein in the PCR product.

**Primer Design.** In the first cycle, the forward and reverse primers were fixed at the same length. This was due to the ease at which Primer3 [103] could be used. The input file was all gene sequences for which no signal peptide sequence was detected (see above). For the second cycle, the prim.aux file was manually inspected. This file contained information about successful primer picks where only a left or right primer could be picked. The strategy was to combine primers of different length. The potential for primer-dimer formation using this strategy was also assessed.

***N.g.* ORF cloning.** Primers for each of the genes were purchased from Illumina, Inc (San Diego, CA) and were designed to add sequences corresponding to part of the *attB* site necessary for recombination into the Gateway® entry vector, pDONR221 (Invitrogen, Carlsbad, CA). Primary amplification was done using genomic DNA at a concentration of ~10 ng/reaction and gene specific primers at 2.5 μM. Reaction mix contained dNTPs, reaction buffer, MgCl_2_ and Taq polymerase as recommended by the manufacturer (Roche). Reactions conditions for primary PCR were as follows: denaturation at 94ºC for 10 min; 10 cycles of 94ºC 30 sec, 50ºC 1 min, 74ºC 1-5 min (depending on length of predicted product); 20 cycles of 94ºC 30 sec, 55ºC 1 min, 74ºC 1-5 min (depending on length of predicted product); and a final extension at 74ºC for 10 min. Following the primary amplification with the primer set, products were diluted 1:100 and 1 μl used as template for a secondary amplification using a pair of primers corresponding to the partial *attB* site common to all of the amplicons, and including additional sequences to generate a complete *attB* site for the cloning reaction. Reactions conditions for secondary PCR were as follows: denaturation at 94ºC for 1 min; 5 cycles of 94ºC 15 sec, 45ºC 30 sec, 68ºC 2 min; 15 cycles of 94ºC 15 sec, 55ºC 30 sec, 68ºC 2 min. 5 μl of the 50 μl PCR was removed and used for cloning, and the remainder used for agarose gel analysis and printing of DNA arrays (*N.g.* array version 1.0, see below). Cloning reactions were performed according to the manufacturer’s instructions (Invitrogen), transformed into *E. coli* strain DH5α, and transformants selected for kanamycin resistance (Kan^R^). Individual transformants were picked into wells of 96-well plates containing 100 μl LB Kan (50 mg/l) broth and the same toothpick then used to place a small amount of bacteria directly into another plate containing a PCR cocktail. PCR was done using the M13 universal primers, which flank the *att* sites of the entry vector, pDONR221, as recommended in the Gateway® manual. A product of 350 bp was observed if no insert was present, providing an internal control for the PCR reactions. Individual clones were identified, stocked in duplicate, and grown for DNA isolation for sequencing.

**Sequencing.** Sequencing runs from each end of the insert of each of the clones was determined to verify the ORF inserted. Complete sequence verification, (ie. both strands completely across the insert) was not done as it was determined to be impractical. DNA sequencing reactions were performed at the Baylor College of Medicine HGSC. Sequence information on the clones is available upon request.

**DNA microarray construction.** *N.g.* array version 1.0 consists of 2033 of the annotated protein coding genes of the *N. gonorrhoeae* genome [15], plus two small RNAs. PCR amplicons remaining from the cloning reactions (described above) were concentrated using Multiscreen 384-well filter plates (Millipore, Billerica, MA) and eluted in 20 μl TE buffer (10 mM Tris, 1 mM EDTA pH 8.0). Samples were then transferred to 384-well polystyrene plates and evaporated to dryness. DNA was resuspended in 10 μl of 3X SSC, stored overnight at 4°C, and then vortexed for 20 minutes to ensure complete suspension. The PCR products were then spotted in duplicate onto TeleChem SuperAmine glass slides (TeleChem International, Inc., Sunnyvale, CA) using a GeneMachines Omnigrid 100 (GeneMachines, Inc., San Carlos, CA) with 16 TeleChem Chipmaker 2 pins in duplicate. Printing of microarray slides was done at the Genome Technology Support Facility (GTSF) at Michigan State University.

**RNA isolation and labelling.** *N. gonorrhoeae* strains were grown in GC broth with supplements [104] and 0.042% sodium bicarbonate to A_600_ of 0.6. 10 mls (2 x 10^8^ CFU) of bacteria were harvested and RNA isolated using Trizol reagent (Invitrogen). RNA was quantified spectrophotometrically, and quality analyzed by agarose gel electrophoresis. DNA contamination of RNA samples was assessed by PCR prior to reverse-transcription. 10 μg of RNA was labelled with either Cy3 or Cy5 using the CyScribe first-strand labelling kit (Amersham Biosciences, Piscataway, NJ) which employs randomly primed reverse transcription in the presence of Cy3 or Cy5-dCTP. Following the labelling reaction, RNA was removed by treatment with NaOH (0.025 M final concentration), and the cDNA purified using the Qiagen PCR cleanup kit, eluting in 20 μl H_2_O.

**Hybridizations.** DNA arrays slides were first UV-crosslinked at 60 mJ energy, and then rinsed twice in 0.1% SDS with vigorous initial agitation to remove the unbound DNA. Slides were then rinsed twice in H_2_O for 2 min each at room temperature. Double-stranded DNA on the slide was denatured by immersion in boiling H_2_O for 3 min, followed by plunging briefly into room temperature H_2_O to cool. Slides were dried by centrifugation for 1 min at 500 x g in a 50 ml conical tube. Slides were prehybridized by spotting 80 μl pre-warmed prehybridization buffer (5X SSC, 0.1% SDS, 1% BSA) onto the array, covering with a large coverslip, and assembling in a Corning hybridization chamber (Corning Life Sciences, Acton, MA). Slides were pre-hybridized at 42°C for ≥ 2 hrs. Cy3- and Cy5-labelled probes were mixed (10 μl each) with 1 μl 1 mg/ml sheared, sonicated herring sperm DNA and denatured by boiling for 5 min, followed by chilling on ice. 20 μl 4X hybridization buffer (Amersham) and 40 μl formamide were added to the denatured DNA and the entire hybridization mix pipetted onto the prehybridized array slide. A new coverslip was placed over the array, the hybridization chamber reassembled, and the array hybridized overnight at 42°C. Following hybridization, the coverslip was removed by gently immersing the slide in the first wash (1X SSC; 0.2% SDS). Subsequent washes were: once in 1X SSC; 0.2% SDS at 42°C, twice in 0.1X SSC; 0.2% SDS at room temperature, once in 0.1X SSC. Slides were dried by centrifugation and scanned using a GenePix 4000B scanner (Axon Instruments, Union City, CA). Images were processed and analyzed using GenePix 4.0 software.

**Gene expression analysis.** pDONR221 derivatives chosen from the clone set were recombined with the destination vector, pDEST15 (Invitrogen), an N-terminal GST fusion vector. Plasmid DNA from the pDONR221 derivatives was isolated and incubated with pDEST15 in a LR recombination reaction performed according to the manufacturer’s instructions. The recombination mixes were transformed into *E. coli* DH5α and transformants were selected on LB plates containing 50 mg/l Carbenicillin (Cb^50^). To test the possibility of false positive clones, the overexpression clones were tested for growth on chloramphenicol (20 mg/l), on which expression recombinants should not grow since the chloramphenicol resistance gene of pDEST15 is replaced by the insert. The resulting plasmids were purified and transformed into *E. coli* BL21-AI (Invitrogen) which expresses T7 RNA polymerase from the *araBAD* promotor, and transformants were selected on LB Cb^50^ plates. BL21-AI strains containing the pDEST15 derivatives were grown overnight and used to inoculate fresh LB medium containing Cb^50^ to an OD_600_ of 0.05. Expression of the GST-fusion proteins in *E. coli* BL21-AI was induced at an OD_600_ of 0.5 by addition of L-arabinose to a final concentration of 0.2%. Aliquots were removed after 2 hr and total proteins electropheresed on 10% polyacrylamide SDS gels [105].

**AUTHORS CONTRIBUTIONS**

Thomas Brettin: design of clone set construction, primer design, annotation of *N. gonorrhoeae* genome sequence

Michael R. Altherr: design of clone set construction, design and supervision of high-throughput PCR amplification of ORFs and cloning into Gateway entry vectors (Figure 1)

Ying Du: all DNA microarray experiments (Tables 1-3), amplification and cloning of several ORFs not obtained in the initial batch cloning

Roxie M. Mason: cataloging of all cloning reactions processed at MSU (Arvidson lab), screening of transformants for clones and preparation of clones sent to Baylor for sequencing

Alexandra Friedrich: recombination of selected clones into tagged, expression vectors and analysis of proteins produced (Figure 2)

Laura Potter: participated in initial project design, screening and cataloging of all cloning reactions processed at OHSU (So lab), screening of transformants for clones and preparation of clones sent to Baylor for sequencing

Chris Langford: batch BLAST analysis of sequencing results

Thomas J. Keller: batch BLAST analysis of sequencing results, wrote new program to faciliate analysis of sequence data

Jason Jens: processing of sequence data from Baylor, batch program design for analysis of subset of clones from Gonococcal Genetic Island

Heather Howie: screening of transformants for clones and preparation of clones sent to Baylor for sequencing

Nathan J. Weyand: screening of transformants for clones and preparation of clones sent to Baylor for sequencing

Susan Clary: screening of transformants for clones and preparation of clones sent to Baylor for sequencing, amplification and cloning of several ORFs not obtained in the initial batch cloning

Kimberly Prichard: primer design

Susi Wachocki: high-throughput PCR amplification of ORFs and cloning into Gateway entry vectors

Erica Sodergren: sequencing of clone set

Joseph P. Dillard: contributed unpublished data of the Gonococcal Genetic Island, participated in project design

George Weinstock: initial project design

Magdalene So: initial project design, supervison of all work performed at OHSU, substantial writing of manuscript

Cindy Grove Arvidson: initial project design, supervison of all work performed at MSU, screening of transformants from LANL, cloning of several ORFs not cloned in initial high-throughput cloning, collated and organanized all sequence data, primary writing of manuscript, corresponding author

**ACKNOWLEDGEMENTS**

Work in the Arvidson laboratory was supported by start-up funds to C.G.A. from the Colleges of Human and Osteopathic Medicine at Michigan State University, and an Intramural Research Grant from the MSU Vice President for Research and Graduate Studies. Work performed at the Los Alamos National Laboratory was supported by the Molecular Foundations of Pathogenesis project, U.S. Department of Energy Contract W-7405-ENG-36 and by funds from the LANL Laboratory Directed Research Development Program to M.R.A. Work in the So laboratory was supported in part by NIH grants AI34560, AI47260 and AI49973 to M. S. Alexandra Friedrich was supported by a fellowship within the Postdoc-Programme of the German Academic Exchange Service (DAAD).

The authors are extremely grateful to thank Jeffrey Landgraf of the Michigan State University GTSF for help and advice in the production of DNA arrays, and Robert Britton of the MSU Department of Microbiology and Molecular Genetics for the use of his GenePix array scanner and many helpful discussions of the use and analysis of DNA arrays. We also thank Audrey Butcher and Catherine Beauduy (Arvidson laboratory) for their participation in analyzing potential clones for the clone set and Anita Amin, Judy Hernandez, Donna Muzny, and the Baylor College of Medicine Human Genome Sequencing Center for assitance in sequencing the clone set.

**REFERENCES**

1.	**Centers for Disease Control and Prevention** [http://www.cdc.gov]

2.	**World Health Organization**. [http://www.who.org]

3.	Fox KK, Knapp JS, Holmes KK, Hook EW 3rd, Judson FN, Thompson SE, Washington JA, Whittington WL: **Antimicrobial resistance in *Neisseria gonorrhoeae* in the United States, 1988-1994: the emergence of decreased susceptibility to the fluoroquinolones.** *J Infect Dis* 1997, **175**:1396-1403.

4.	Shlaes D, Levy, S, and Archer, G: **Antimicrobial resistance: new directions.** *ASM News* 1991, **57**:455-458.

5.	Schneider H, Hale, TL, Zollinger, WD, Seid, RC Jr., Hammack, CA, and Griffiss, JM: **Does an experimental gonococcal infection protect human volunteers from subsequent reinfection?** *Abstracts of the Tenth International Pathogenic Neisseria Conference* 1996, **10**:13.

6.	Laga M, Manoka A, Kivuvu M, Malele B, Tuliza M, Nzila N, Goeman J, Behets F, Batter V, Alary M: **Non-ulcerative sexually transmitted diseases as risk factors for HIV-1 transmission in women: results from a cohort study [see comments]**. *AIDS* 1993, **7**:95-102.

7.	Fletcher JD, Stratton J, Chandler CS, Sparling PF: **Epidemiologic notes and reports penicillin-resistant gonorrhea -- North Carolina.** *Morbidity and Mortality Weekly Report* 1983, **32**:273-275.

8.	Turner CF, Rogers SM, Miller HG, Miller WC, Gribble JN, Chromy JR, Leone PA, Cooley PC, Quinn TC, Zenilman JM: **Untreated gonococcal and chlamydial infection in a probability sample of adults.** *JAMA* 2002, **287**:726-733.

9.	Hauck CR, Meyer TF: **'Small' talk: Opa proteins as mediators of *Neisseria*-host-cell communication.** *Curr Opin Microbiol* 2003, **6**:43-49.

10.	Merz AJ, So M: **Interactions of pathogenic *Neisseriae* with epithelial cell membranes**. *Annu Rev Cell Dev Biol* 2000, **16**:423-457.

11.	Pujol C, Eugene E, Morand P, Nassif X: **Do pathogenic neisseriae need several ways to modify the host cell cytoskeleton?** *Microbes Infect* 2000, **2**:821-827.

12.	McKevitt M, Patel K, Smajs D, Marsh M, McLoughlin M, Norris SJ, Weinstock GM, Palzkill T: **Systematic cloning of *Treponema pallidum* open reading frames for protein expression and antigen discovery.** *Genome Res* 2003, **13**:1665-1674.

13.	Nicholson TL, Olinger L, Chong K, Schoolnik G, Stephens RS: **Global stage-specific gene regulation during the developmental cycle of *Chlamydia trachomatis*.** *J Bacteriol* 2003, **185**:3179-3189.

14.	Walhout AJ, Temple GF, Brasch MA, Hartley JL, Lorson MA, van den Heuvel S, Vidal M: **GATEWAY recombinational cloning: application to the cloning of large numbers of open reading frames or ORFeomes.** *Meth Enzymol* 2000, **328**:575-592.

15.	***Neisseria gonorrhoeae* annotated genome sequence database.** [http://www.stdgen.lanl.gov]

16.	Dillard JP, Seifert HS: **A variable genetic island specific for *Neisseria gonorrhoeae* is involved in providing DNA for natural transformation and is found more often in disseminated infection isolates.** *Mol Microbiol* 2001, **41**(1):263-277.

17.	Hamilton HL, Dominguez NM, Schwartz KJ, Hackett KT, Dillard JP: ***Neisseria gonorrhoeae* secretes chromosomal DNA via a novel type IV secretion system.** *Mol Microbiol* 2005, **in press**.

18.	**STADEN DNA sequence analysis package** [http://staden.sourceforge.net]

19.	Altschul SF, Madden TL, Schäffer AA, Zhang J, Zhang Z, Miller W, and Lipman, DJ: **Gapped BLAST and PSI-BLAST: a new generation of protein database search programs.** *Nucleic Acids Res* 1997, **25**:3389-3402.

20.	Tatusova TA, Madden TL: **Blast 2 sequences - a new tool for comparing protein and nucleotide sequences**. *FEMS Microbiol Lett* 1999, **174**:247-250.

21.	Frasz C, Arvidson CG: **A role for both DNA and RNA in GTP hydrolysis by the *Neisseria gonorrhoeae* signal recognition particle receptor.** *J Bacteriol* 2003, **185**:801-808.

22.	Gueneau de Novoa P, Williams KP: **The tmRNA website: reductive evolution of tmRNA in plastids and other symbionts.** *Nucleic Acids Res* 2004, **32**:D104-108.

23.	Swanson J, Kraus SJ, Gotschlich EC: **Studies on gonococcus infection. I. Pili and zones of adhesion: their relation to gonococcal growth patterns**. *J Exp Med* 1971, **134**:886-906.

24.	Meyer TF, Billyard E, Haas R, Storzbach S, So M: **Pilus genes of *Neisseria gonorrheae*: chromosomal organization and DNA sequence**. *Proc Natl Acad Sci USA* 1984, **81**:6110-6114.

25.	Klimpel KW, Clark VL: **Multiple protein differences exist between *Neisseria gonorrhoeae* type 1 and type 4.** *Infect Immun* 1988, **56**:808-814.

26.	Merz AJ, Rifenbery DB, Arvidson CG, So M: **Traversal of a polarized epithelium by pathogenic Neisseriae: facilitation by type IV pili and maintenance of epithelial barrier function**. *Mol Med* 1996, **2**:745-754.

27.	Britton RA, Eichenberger P, Gonzalez-Pastor JE, Fawcett P, Monson R, Losick R, Grossman AD: **Genome-wide analysis of the stationary-phase sigma factor (sigma-H) regulon of *Bacillus subtilis*.** *J Bacteriol* 2002, **184**:4881-4890.

28.	Seifert HS: **Questions about gonococcal pilus phase- and antigenic variation**. *Mol Microbiol* 1996, **21**:433-440.

29.	Koomey M, Gotschlich EC, Robbins K, Bergstrom S, Swanson J: **Effects of *recA* mutations on pilus antigenic variation and phase transitions in *Neisseria gonorrhoeae***. *Genetics* 1987, **117**:391-398.

30.	Mehr IJ, Seifert HS: **Random shuttle mutagenesis: gonococcal mutants deficient in pilin antigenic variation**. *Mol Microbiol* 1997, **23**:1121-1131.

31.	Hagblom P, Segal E, Billyard E, So M: **Intragenic recombination leads to pilus antigenic variation in *Neisseria gonorrhoeae***. *Nature* 1985, **315**:156-158.

32.	Segal E, Hagblom P, Seifert HS, So M: **Antigenic variation of gonococcal pilus involves assembly of separated silent gene segments**. *Proc Natl Acad Sci USA* 1986, **83**:2177-2181.

33.	Merz AJ, So M, Sheetz MP: **Pilus retraction powers bacterial twitching motility.** *Nature* 2000, **407**:98-102.

34.	Wolfgang M, van Putten JPM, Hayes SF, Dorward D, Koomey M: **Components and dynamics of fiber formation define a ubiquitous biogenesis pathway for bacterial pili.** *EMBO J* 2000, **19**:6408-6418.

35.	Fischer SH, Rest RF: **Gonococci possessing only certain P.II outer membrane proteins interact with human neutrophils.** *Infect Immun* 1988, **56**:1574-1579.

36.	Jonsson AB, Nyberg G, Normark S: **Phase variation of gonococcal pili by frameshift mutation in *pilC*, a novel gene for pilus assembly**. *EMBO J* 1991, **10**:477-488.

37.	Lambden P, Robertson J, Watt P: **Biological properties of two distinct pilus types produced by isogenic variants of *Neisseria gonorrhoeae* P9**. *J Bacteriol* 1980, **141**:393-396.

38.	Makino S, van Putten JP, Meyer TF: **Phase variation of the opacity outer membrane protein controls invasion by *Neisseria gonorrhoeae* into human epithelial cells**. *EMBO J* 1991, **10**:1307-1315.

39.	Punsalang AP, Sawyer WD: **Role of pili in the virulence of *Neisseria gonorrhoeae***. *Infect Immun* 1973, **8**:255-263.

40.	Rudel T, Scheurerpflug I, Meyer TF: ***Neisseria* PilC protein identified as type-4 pilus tip-located adhesin**. *Nature* 1995, **373**:357-359.

41.	Swanson J: **Studies on gonococcus infection. IV. Pili: their role in attachment of gonococci to tissue culture cells**. *J Exp Med* 1973, **137**:571-589.

42.	Swanson J, Robbins K, Barrera O, Corwin D, Boslego J, Ciak J, Blake M, Koomey JM: **Gonococcal pilin variants in experimental gonorrhea**. *J Exp Med* 1987, **165**:1344-1357.

43.	van Putten JP, Duensing TD, Carlson J: **Gonococcal invasion of epithelial cells driven by P.IA, a bacterial ion channel with GTP binding properties**. *J Exp Med* 1998, **188**:941-952.

44.	Waldbeser LS, Ajioka RS, Merz AJ, Puaoi D, Lin L, Thomas M, So M: **The *opaH* locus of *Neisseria gonorrhoeae* MS11A is involved in epithelial cell invasion**. *Mol Microbiol* 1994, **13**:919-928.

45.	Weel GFL, Hopman CTP, Van Putten JPM: **In situ expression and localization of *Neisseria gonorrhoeae* opacity proteins in infected epithelial cells: apparent role of Opa proteins in cellular invasion**. *J Exp Med* 1991, **173**:1395-1405.

46.	Schryvers AB, Stojiljkovic I: **Iron acquisition systems in the pathogenic *Neisseria.*** *Mol Microbiol* 1999, **32**:1117-1123.

47.	Lin L, Ayala P, Larson J, Mulks M, Fukuda M, Carlsson SR, Enns C, So M: **The *Neisseria* type 2 IgA1 protease cleaves LAMP1 and promotes survival of bacteria within epithelial cells**. *Mol Microbiol* 1997, **24**:1083-1094.

48.	Simpson DA, Hausinger RP, Mulks M: **Purification, characterization, and comparison of the immunoglobulin A1 proteases of *Neisseria gonorrhoeae*.** *J Bacteriol* 1988, **170**:1866-1873.

49.	Gregg CR, Melly MA, Hellerqvist CG, Coniglio JG, McGee ZA: **Toxic activity of purified lipopolysaccharide of *Neisseria gonorrhoeae* for human fallopian tube mucosa**. *J Infec Dis* 1981, **143**:432-439.

50.	Griffiss JM, Schneider H, Mandrell RE, Jarvis GA, Kim JJ, Gibson B, Apicella MA: **The immunochemistry of neisserial LOS.** *Antonie Van Leeuwenhoek* 1987, **53**:501-507.

51.	Griffiss J, Schneider H, Mandrell R, Yamasaki R, Jarvis G, Kim J, Gibson B, Hamadeh R, Apicella M: **Lipooligosaccharides: the principal glycolipids of the Neisserial outer membrane**. *Rev Infect Dis* 1988, **10**:S287-S295.

52.	Mandrell R, Schneider H, Apicella M, Zollinger W, Rice PA, Griffis JM: **Antigenic and physical diversity of *Neisseria gonorrhoeae* lipooligosaccharides.** *Infect Immun* 1986, **54**:63-69.

53.	Melly MA, McGee ZA, Rosenthal RS: **Ability of monomeric peptidoglycon fragments from *Neisseria gonorrhoeae* to damage human fallopian-tube mucosa**. *J Infec Dis* 1984, **149**:378-386.

54.	Schneider H, Hale T, Zollinger W, Seid RJ, Hammack C, Griffiss J: **Heterogeneity of molecular size and antigenic expression within lipooligosaccharides of individual strains of *Neisseria gonorrhoeae* and *Neisseria meningitidis***. *Infect Immun* 1984, **45**:544-549.

55.	Chen T, Gotschlich EC: **CGM1a antigen of neutrophils, a receptor of gonococcal opacity proteins**. *Proc Natl Acad Sci USA* 1996, **93**:14851-14856.

56.	Dehio M, Gomez-Duarte OG, Dehio C, Meyer TF: **Vitronectin-dependent invasion of epithelial cells by *Neisseria gonorrhoeae* involves alpha(v) integrin receptors**. *FEBS Lett* 1998, **424**:84-88.

57.	Duensing TD, van Putten JP: **Vitronectin mediates internalization of *Neisseria gonorrhoeae* by Chinese hamster ovary cells**. *Infect Immun* 1997, **65**:964-970.

58.	Gomez-Duarte OG, Dehio M, Guzman CA, Chhatwal GS, Dehio C, Meyer TF: **Binding of vitronectin to opa-expressing *Neisseria gonorrhoeae* mediates invasion of HeLa cells**. *Infect Immun* 1997, **65**:3857-3866.

59.	Kallstrom H, Liszewski MK, Atkinson JP, Jonsson AB: **Membrane cofactor protein (MCP or CD46) is a cellular pilus receptor for pathogenic *Neisseria***. *Mol Microbiol* 1997, **25**:639-647.

60.	Spence JM, Clark VL: **Role of ribosomal protein L12 in gonococcal invasion of Hec1B cells.** *Infect Immun* 2000, **68**:5002-5010.

61.	van Putten JP, Paul SM: **Binding of syndecan-like cell surface proteoglycan receptors is required for *Neisseria gonorrhoeae* entry into human mucosal cells**. *EMBO J* 1995, **14**:2144-2154.

62.	van Putten JP, Duensing TD, Cole RL: **Entry of OpaA+ gonococci into HEp-2 cells requires concerted action of glycosaminoglycans, fibronectin and integrin receptors**. *Mol Microbiol* 1998, **29**:369-379.

63.	Virji M, Evans D, Hadfield A, Grunert F, Teixeira AM, Watt SM: **Critical determinants of host receptor targeting by *Neisseria meningitidis* and *Neisseria gonorrhoeae*: identification of Opa adhesiotopes on the N-domain of CD66 molecules**. *Mol Microbiol* 1999, **34**:538-551.

64.	Chen JCR, Bavoil P, Clark VL: **Enhancement of the invasive ability of *Neisseria gonorrhoeae* by contact with Hec1B, an adenocarcinoma endometrial cell line**. *Mol Microbiol* 1991, **5**:1531-1538.

65.	Ayala P, Lin L, Hopper S, Fukuda M, So M: **Infection of epithelial cells by pathogenic neisseriae reduces the levels of multiple lysosomal constituents**. *Infect Immun* 1998, **66**:5001-5007.

66.	Ayala BP, Vasquez B, Clary S, Tainer JA, Rodland K, So M: **The pilus-induced Ca^2+^ flux triggers lysosome exocytosis and increases the amount of Lamp1 accessible to *Neisseria* IgA1 protease**. *Cell Microbiol* 2001, **3**:265-275.

67.	Kallstrom H, Islam MS, Berggren PO, Jonsson AB: **Cell signaling by the type IV pili of pathogenic Neisseria**. *J Biol Chem* 1998, **273**:21777-21782.

68.	Merz AJ, Enns CA, So M: **Type IV pili of pathogenic neisseriae elicit cortical plaque formation in epithelial cells**. *Mol Microbiol* 1999, **32**:1316-1332.

69.	Chen T, Grunert F, Medina-Marino A, Gotschlich EC: **Several carcinoembryonic antigens (CD66) serve as receptors for gonococcal opacity proteins**. *J Exp Med* 1997, **185**:1557-1564.

70.	Gray-Owen SD, Dehio C, Haude A, Grunert F, Meyer TF: **CD66 carcinoembryonic antigens mediate interactions between Opa- expressing *Neisseria gonorrhoeae* and human polymorphonuclear phagocytes**. *EMBO J* 1997, **16**:3435-3445.

71.	Lee SW, Bonnah RA, Higashi DL, Atkinson JP, Milgram SL, So M: **CD46 is phosphorylated at tyrosine 354 upon infection of epithelial cells by *Neisseria gonorrhoeae*.** *J Cell Biol* 2002, **156**:651-657.

72.	Sperandio V, Torres AG, Jarvis B, Nataro JP, Kaper JB: **Bacteria-host communication: the language of hormones**. *Proc Natl Acad Sci USA* 2003, **100**:8951-8956.

73.	Binnicker MJ, Williams RD, Apicella MA: **Gonococcal porin IB activates NF-kappaB in human urethral epithelium and increases the expression of host antiapoptotic factors.** *Infect Immun* 2004, **72**:6408-6417.

74.	Roe BA, Lin SP, Song L, Yuan X, Clifton S, Dyer D: **Gonococcal genome sequencing project. URL http://www.genome.ou.edu/gono/html**. 2002.

75.	Du Y, Arvidson CG: **Identification of ZipA, an SRP-targeted protein from *Neisseria gonorrhoeae.*** *J Bacteriol* 2003, **185**:2122-2130.

76.	Seifert HS, Ajioka RS, Paruchuri D, Heffron F, So M: **Shuttle mutagenesis of *Neisseria gonorrhoeae*: pilin null mutations lower DNA transformation competence**. *J Bacteriol* 1990, **172**:40-46.

77.	Goodman SD, Scocca JJ: **Identification and arrangement of the DNA sequence recognized in specific transformation of *Neisseria gonorrhoeae***. *Proc Natl Acad Sci USA* 1988, **85**:6982-6986.

78.	Dempsey JF, Litaker W, Hadhure A, Snodgrass TL, Cannon JG: **Physical map of the chromosome of *Neisseria gonorrhoeae* FA1090 with locations of genetic markers, including *opa* and *pil* genes.** *J Bacteriol* 1991, **173**:5476-5486.

79.	Cotter PA, Miller JF: ***In vivo* and ex vivo regulation of bacterial virulence gene expression.** *Curr Opin Microbiol* 1998, **1**:17-26.

80.	Dorman CJ, Porter ME: **The *Shigella flexneri* virulence gene regulatory cascade: A paradigm of bacterial control mechanisms.** *Mol Microbiol* 1998, **29**:677-684.

81.	Groisman EA, Chiao E, Lipps CJ, Heffron F: ***Salmonella typhimurium phoP* virulence gene is a transcriptional regulator.** *Proc Natl Acad Sci USA* 1989, **86**:7077-7081.

82.	Lucas RL, Lee CA: **Unravelling the mysteries of virulence gene regulation in *Salmonella typhimurium*.** *Mol Microbiol* 2000, **36**:1024-1033.

83.	Miller SI, Kukral AM, Mekalanos JJ: **A two-component regulatory system (*phoP phoQ*) controls *Salmonella typhimurium* virulence.** *Proc Natl Acad Sci USA* 1989, **86**:5054-5058.

84.	Pettersson J, Nordfelth R, Dubinina E, Bergman T, Gustafsson M, Magnusson KE, Wolf-Watz H: **Modulation of virulence factor expression by pathogen target cell contact.** *Science* 1996, **273**:1231-1233.

85.	Skorupski K, Taylor RK: **Control of the ToxR virulence regulon in *Vibrio cholerae* by environmental stimuli.** *Mol Microbiol* 1997, **25**:1003-1009.

86.	Sebastian S, Agarwal S, Murphy JR, Genco CA: **The gonococcal *fur* regulon: identification of additional genes involved in major catabolic, recombination, and secretory pathways.** *J Bacteriol* 2002, **184**:3965-3974.

87.	West S, Sparling F: **Response of *Neisseria gonorrhoeae* to iron limitation: alterations in expression of membrane proteins without apparent siderophore production.** *Infect Immun* 1985, **47**:388-394.

88.	Hagman KE, Shafer WM: **Transcriptional control of the *mtr* efflux system of *Neisseria gonorrhoeae*.** *J Bacteriol* 1995, **177**:4162-4165.

89.	Lee EH, Rouquette-Loughlin C, Folster JP, Shafer WM: **FarR regulates the *farAB*-encoded efflux pump of *Neisseria gonorrhoeae* via an MtrR regulatory mechanism.** *J Bacteriol* 2003, **185**:7145-7152.

90.	Cummings CA, Relman DA: **Using DNA microarrays to study host-microbe interactions.** *Emerg Infect Dis* 2000, **6**:513-525.

91.	Grifantini R, Sebastian S, Frigimelica E, Draghi M, Bartolini E, Muzzi A, Rappuoli R, Grandi G, Genco CA: **Identification of iron-activated and -repressed Fur-dependent genes by transcriptome analysis of *Neisseria meningitidis* group B.** *Proc Natl Acad Sci USA* 2003, **100**:9542-9547.

92.	Guckenberger M, Kurz S, Aepinus C, Theiss S, Haller S, Leimbach T, Panzner U, Weber J, Paul H, Unkmeir A *et al*: **Analysis of the heat shock response of *Neisseria meningitidis* with cDNA and oligonucleotide-based DNA microarrays.** *J Bacteriol* 2002, **184**:2546-2551.

93.	Dietrich G, Kurz S, Hübner C, Aepinus C, Theiss S, Guckenberger M, Panzner U, Weber J, Frosch M: **Transcriptome analysis of *Neisseria meningitidis* during infection.** *J Bacteriol* 2003, **185**:155-164.

94.	Grifantini R, Bartolini E, Muzzi A, Draghi M, Frigimelica E, Berger J, Randazzo F, Grandi G: **Gene expression profile in *Neisseria meningitidis* and *Neisseria lactamica* upon host-cell contact.** *Ann NY Acad Sci* 2002, **975**:202-216.

95.	Seifert HS: **Insertionally inactivated and inducible *recA* alleles for use in *Neisseria* [published erratum appears in Gene 1997 197:415]**. *Gene* 1997, **188**:215-220.

96.	Shaw JH, Falkow S: **Model for invasion of human tissue culture cells by *Neisseria gonnorrhoeae***. 1988, **56**:1625-1632.

97.	McGee ZA, Johnson AP, Taylor-Robinson D: **Human fallopian tubes in organ culture: preparation, maintenance, and quantitation of damage by pathogenic microorganisms.** *Infect Immun* 1976, **13**:608-618.

98.	McGee ZA, Johnson AP, Taylor-Robinson D: **Pathogenic mechanisms of *Neisseria gonorrhoeae*: observations on damage to human Fallopian tubes in organ culture by gonococci of colony type 1 or type 4**. *J Infect Dis* 1981, **143**:413-422.

99.	McGee Z, Stephens D, Hoffman L, Schlech III W, Horn R: **Mechanisms of mucosal invasion by pathogenic *Neisseria***. *Rev Infect Dis* 1983, **5**:S708-S714.

100.	Jerse AE: **Experimental gonococcal genital tract infection and opacity protein expression in estradiol-treated mice**. *Infect Immun* 1999, **67**:5699-5708.

101.	Nakai K, Kanehisa M: **Expert system for predicting localization sites in Gram-negative bacteria.** *PROTEINS: Structure, Function, and Genetics* 1991, **11**:95-110.

102.	Nielsen H, Engelbrecht J, Brunak S, von Heijne G: **Identification of prokaryotic and eukaryotic signal peptides and prediction of their cleavage sites.** *Protein Engineering* 1997, **10**:1-6.

103.	Rozen S, Skaletsky H: **Primer3 on the WWW for general users and for biologist programmers.** In: *Bioinformatics Methods and Protocols.* Edited by Krawetz SaM, S.: Humana Press, Totowa, NJ; 2000: 365-386.

104.	Kellogg DS, Peacock WL, Deacon WE, Brown L, Pirkle CI: ***Neisseria gonorrhoeae.* I. Vvirulence genetically linked to clonal varation**. *J Bacteriol* 1963, **85**:1274-1279.

105.	Laemmli UK: **Cleavage of structural proteins during the assembly of the head of bacteriophage T4.** *Nature* 1970, **227**:680-685.

**FIGURE LEGENDS**

**Figure 1.** Flow diagram of the process of generating the *N. gonorrhoeae* clone set and associated DNA microarrays. Steps from primer selection to Gateway® cloning were performed at Los Alamos National Laboratories. Transformation reactions were then divided and one half shipped to Michigan State University (MSU) and the other half to Oregon Health and Sciences University (OHSU) for screening of transformants. Once positive clones were identified, they were arranged in a new 96-well format and replicates shipped to the Baylor HGSC for DNA sequencing. Copies of sequencing plates were sent to MSU and OHSU such that each institution has a copy of the entire clone set. Success rates are indicated at each step and described in the text.

**Figure 2.** SDS-PAGE analysis of NG ORF-GST fusions. Equivalent amounts of total protein was boiled in sample buffer and electrophoresed on 10% polyacrylamide gels. Gels were stained with Coomassie Blue. Lane 1: MW markers; lane 2: NG1490 uninduced; lane 3: NG1490 induced (38.6 kdal); lane 4: NG1561 uninduced; lane 5: NG1561 induced (58.0 kdal); lane 6: NG1641 uninduced; lane 7: NG1641 induced (65.2 kdal).

**^TABLE 1. Genes increased in expression in P^**+

| **ORF ID** | ***gene*** | **gene product** | **P^+^/P^-^ ave** |
| --- | --- | --- | --- |
| NG2061 | *pilE* | pilin expression locus | 67.13 |
| NG1512.1 | *pilS* | silent pilin locus | 15.09 |
| NG2062 | *pilS* | silent pilin locus | 12.04 |
| NG3002 | *pilS* | silent pilin locus | 6.58 |
| NG0795 | *bfrB* | bacterioferritin | 4.04 |
| NG0794 | *bfrA* | bacterioferritin | 3.79 |
| NG5001 | *traD* | type IV secretion component | 3.44 |
| NG1046 | *clpB* | endopeptidase Clp ATP-binding chain B | 2.86 |
| NG0376 | *ppiB* | peptidyl-prolyl cis-trans isomerase B (rotamase B) | 2.82 |
| NG1422 | *grpE* | HSP-70 cofactor; nucleotide exchange factor | 2.77 |
| NG5041 | *ydhB* | hypothetical protein, GGI^a^ | 2.36 |
| NG1668 | *pgi* | glucose-6-phosphate isomerase | 2.32 |
| NG0904 |  | conserved hypothetical protein | 2.26 |
| NG0318 | *recN* | DNA repair protein RecN | 2.18 |
| NG0733 |  | int gene product | 2.12 |
| NG0906 |  | probable iron-sulphur protein | 2.11 |
| NG0905 |  | conserved hypothetical protein | 2.11 |
| NG0634 |  | hypothetical protein | 2.04 |
| NG0084 | *pglC* | pilin glycosylation protein | 2.02 |
| NG1769 | *yhjA* | cytochrome c peroxidase | 2.02 |
| NG0399 | *htpX* | heat shock protein (probable membrane-bound zinc metallopeptidase) | 1.97 |
| NG0375 | *pgm* | phosphoglucomutase | 1.95 |
| NG1633 |  | hypothetical protein | 1.90 |
| NG0305 | *himA* | IHF alpha subunit | 1.89 |
| NG2095 | *groEL* | heat shock protein, 60 Kd subunit | 1.85 |
| NG1901 | *dnaJ* | HSP-40/chaperone DnaJ | 1.80 |
| NG0186.1 |  | IS1016 transposase fragment | 1.78 |

^a^Gonococcal Genetic Island [16, 17].

**TABLE 2. Genes increased in expression in P^-^**

| **ORF ID** | ***gene*** | **comments** | **P^+^/P^-^ ave** | **P^-^/P^+^ ave** |
| --- | --- | --- | --- | --- |
| NG0641 | *mod* | type III restriction/modification system modification methylase | 0.36 | 2.74 |
| NG1294 | *lrp* | leucine-responsive regulatory protein | 0.42 | 2.37 |
| NG1473 | *mdaB* | drug activity modulator B | 0.51 | 1.98 |

**^TABLE 3. Additional genes slightly increased in expression in P^**-

| **ORF ID** | ***gene*** | **gene product** | **P^+^/P^-^ ave** | **P^-^/P^+^ ave** |
| --- | --- | --- | --- | --- |
| NG1676 | *rplU* | 50S ribosomal protein L21 (RplU) | 0.56 | 1.79 |
| NG1837 | *rplD* | 50S ribosomal protein L4 | 0.57 | 1.75 |
| NG1818 | *rpoA* | DNA-directed RNA polymerase α chain (RpoA) | 0.57 | 1.75 |
| NG1822 | *secY* | preprotein translocase SecY | 0.59 | 1.68 |
| NG1836 | *rplW* | 50S ribosomal protein L23 | 0.60 | 1.67 |
| NG0298 | *rplT* | 50S ribosomal protein L20 | 0.60 | 1.66 |
| NG1854 | *rplA* | 50S ribosomal protein L1 | 0.60 | 1.65 |
| NG1825 | *rplF* | 50S ribosomal protein L6 | 0.61 | 1.65 |
| NG1835 | *rplB* | 50S ribosomal protein L2 | 0.61 | 1.64 |
| NG1821 | *rpsM* | 30S ribosomal protein S13 | 0.62 | 1.62 |
| NG1817 | *rplQ* | 50S ribosomal protein L17 | 0.62 | 1.62 |
| NG1823 | *rplO* | 50S ribosomal protein L15 | 0.63 | 1.59 |
| NG1826 | *rpsH* | 30S ribosomal protein S8 | 0.64 | 1.57 |
| NG1657 | *bolA* | BolA/YrbA family protein | 0.64 | 1.57 |
| NG1826.1 | *RS14* | 30S ribosomal protein S14 | 0.64 | 1.55 |
| NG1843 | *fusA* | translation elongation factor EF-G | 0.65 | 1.55 |
| NG0650 | *rhlE* | ATP-dependent RNA helicase | 0.65 | 1.55 |
| NG1855 | *rplK* | 50S ribosomal protein L11 | 0.65 | 1.53 |
| NG1852 | *rplL* | 50S ribosomal protein L7/L12 | 0.66 | 1.52 |
| NG1677 | *rpmA* | 50S ribosomal protein L27 | 0.68 | 1.48 |
| NG1829 | *rplN* | 50S ribosomal protein L14 | 0.68 | 1.46 |
| NG1827 | *rplE* | 50S ribosomal protein L5 | 0.69 | 1.46 |
