# Supplementary material for: Expression capable library for studies of Neisseria gonorrhoeae, version 1.0
Source: BMC Microbiol. 2005 Sep 1;5:50. doi: 10.1186/1471-2180-5-50 (PMC1236931; doi:10.1186/1471-2180-5-50)
Supplement: Additional File 1 — Zipped file which includes all of the sequencing data as .exp files generated from binary data using PREGAP4. These .exp files contain all of the sequences with quality cutoffs included and can be opened with word processing programs. [file 1471-2180-5-50-S1.zip › BMC rebuttal.doc]

Editors
BioMed Central

BMC Microbiology
November 30, 2026

Dear Editors,

Thank you for handling our mansucript entitled “Expression Capable Library for Studies of *Neisseria gonorrhoeae*, version 1.0” by Thomas Brettin *et al*, (Cindy Grove Arvidson, corresponding author). We have read and considered the reviews and made modifications as suggested. We have also included tables of the genes in the clone set (including those whose sequence is validated) and an additional zipped file containing the raw sequence data as supplementary material.

I hope these changes are satisfactory. Please let me know if there is anything else you would like us to modify.

All authors, T. Brettin, M. R. Altherr, Y. Du, R. M. Mason, A. Friedrich, L. Potter, C. Langford, T. J. Keller, J. Jens, H. Howie, N. J. Weyand, S. Clary, K. Prichard, S. Wachocki, E. Sodergren, J. P. Dillard, G. Weinstock, and M. So have agreed to the modifications as written. We appreciate your attention in this matter.

The point-by-point responses are as follows:

Reviewer 1:

Major issues

Point 1. Demonstration of the utility of the Gateway clone set.

Our most frequent use of the clone set is currently to use individual clones as targets for in vitro transposition, followed by transformation into *N. gonorrhoeae* to generate insertion mutants via allelic replacement. A manuscript in which this method is described in in press (Infection and Immunity) and we’ve attached a PDF of the pre-print of this manuscript, and cited it at the appropriate place in the Discussion section. We have also used the recombination system to generate N-terminal His6-tagged versions of two hypothetical proteins of unknown function and have purifed them for structural studies. These studies are not yet complete and are part of a larger study that will be published elsewhere.

Point 2. Request to include the sequence traces from the (partial) sequence validation of the clone set.

Rather than include the actual traces, which might be readible to a limited number of people due to the software, we have added a zipped file which includes all of the sequencing data as .exp files. As stated in the Results section, each individual sequence read was processed using STADEN software, using PREGAP4 to generate text files from the binary data generated. These .exp files contain all of the sequences with quality cutoffs included. They can be opened with word processing programs and the sequences easily copied for BLAST or other analyses. We refer to this as supplementary file 1 in the text.

An additional table (Table X) listing the cloned genes and their sequence status has been added.

2.

Sincerely,

Cindy Grove Arvidson

Assistant Professor

Department of Microbiology and Molecular Genetics

Michigan State University

517-355-6463 x1573

517-353-8957 FAX

[arvidso3@msu.edu](mailto:arvidso3@msu.edu)
